# Characterizing TLR4 agonist EmT4™ as an anti-*Mycobacterium tuberculosis* vaccine adjuvant

**DOI:** 10.1093/immhor/vlaf014

**Published:** 2025-04-24

**Authors:** Sasha E Larsen, Maham Rais, Valerie A Reese, Debora Ferede, Tiffany Pecor, Suhavi Kaur, Deepika Nag, Thomas Smytheman, Sean A Gray, Darrick Carter, Susan L Baldwin, Rhea N Coler

**Affiliations:** Center for Global Infectious Disease Research, Seattle Children's Research Institute, Seattle Children's Hospital, Seattle, WA, United States; Center for Global Infectious Disease Research, Seattle Children's Research Institute, Seattle Children's Hospital, Seattle, WA, United States; Center for Global Infectious Disease Research, Seattle Children's Research Institute, Seattle Children's Hospital, Seattle, WA, United States; Center for Global Infectious Disease Research, Seattle Children's Research Institute, Seattle Children's Hospital, Seattle, WA, United States; Center for Global Infectious Disease Research, Seattle Children's Research Institute, Seattle Children's Hospital, Seattle, WA, United States; Center for Global Infectious Disease Research, Seattle Children's Research Institute, Seattle Children's Hospital, Seattle, WA, United States; Center for Global Infectious Disease Research, Seattle Children's Research Institute, Seattle Children's Hospital, Seattle, WA, United States; Center for Global Infectious Disease Research, Seattle Children's Research Institute, Seattle Children's Hospital, Seattle, WA, United States; PAI Life Sciences Inc, Seattle, WA, United States; PAI Life Sciences Inc, Seattle, WA, United States; Center for Global Infectious Disease Research, Seattle Children's Research Institute, Seattle Children's Hospital, Seattle, WA, United States; Center for Global Infectious Disease Research, Seattle Children's Research Institute, Seattle Children's Hospital, Seattle, WA, United States; Department of Global Health, University of Washington, Seattle, WA, United States; Department of Pediatrics, University of Washington School of Medicine, Seattle, WA, United States

**Keywords:** adjuvant, mouse model, *Mycobacterium tuberculosis*, TB, vaccine

## Abstract

Tuberculosis (TB) is again the deadliest infectious disease globally, and more efficacious vaccines are needed to reduce this mortality. Successful subunit TB vaccines need antigens and adjuvants that are immunogenic, inexpensive, and accessible. Here we evaluated the potential of synthetically produced Monophosphoryl lipid A (SyMLP), a TLR4-agonist, formulated in an oil-in-water emulsion (EmT4™) in combination with selected fusion proteins, to drive an effective vaccine-mediated immunogenic response in C57BL/6 mice against *Mycobacterium tuberculosis* (M.tb) HN878 and H37Rv challenge. We first observed that EmT4™ enhances activation of C57BL/6 bone-marrow derived macrophages and dendritic cells measured by CD40, CD86, and MHCII expression by flow cytometry. EmT4™ did not induce safety signals in a scaled tolerability study. In immunogenicity studies, mice immunized 3 times 3 weeks apart with ID93 antigen + EmT4™ produced a significantly higher magnitude of circulating proinflammatory cytokines and ID93-specific immunoglobulin G (IgG) antibodies pre- and post-challenge with M.tb than saline control animals. *Ex vivo* ID93 restimulated splenocytes and lung cells elicited significant polyfunctional CD4^+^ T-helper 1 responses. Importantly, ID93 + EmT4™ immunizations significantly reduced bacterial burden in C57BL/6 mice 4 weeks post-challenge. Interestingly, EmT4™ paired with a next generation protein fusion ID91 also afforded prophylactic protection against M.tb HN878 challenge in both young (6 to 8 wk) and aged (20 mo) immunocompromised Beige mice. These protection and immunogenicity findings suggest that synthetically derived EmT4™ adjuvant is not only suitable to help backfill the preclinical TB vaccine candidate pipeline but is also suitable for the needs of the global community.

## Introduction

Tuberculosis (TB) continues to be the leading cause of mortality from an infectious pathogen (*Mycobacterium tuberculosis*, M.tb) and has unfortunately remained at the top of this list for 5 of the last 10 years. Recent progress against TB disease was muted due to pandemic-related setbacks, where 10.6 million people fell ill in 2021 and 2022, and total deaths were reported as 1.25 million in the 2024 Global TB Report.^1–3^ Whereas there are options for antimicrobial drug treatment for clinical or suspected TB disease, bacillus Calmette–Guérin (BCG) remains the only licensed vaccine against M.tb. Furthermore, the efficacy of BCG is well documented to wane after 10 years’ time; therefore, efforts to develop more effective vaccines are urgently needed.^4^^,^^5^ Although BCG and drug treatment alone are not enough to stop the TB pandemic, novel vaccines which boost BCG and/or target prevention of M.tb infection or TB disease could change the tide in this fight. Mathematical modeling predicts that efficacious vaccines could reduce loss of life and morbidity but also significantly affect the overall cost endured by TB control measures. TB vaccines are likely to be highly cost-effective, helping to advance health equity partly by improving economic growth as described by the World Health Organization (WHO) Global Tuberculosis Programme in 2022 “An investment case for new tuberculosis vaccines.”^6^ Indeed, it is estimated that a 50% effective prevention of disease (POD) vaccine targeting adolescent and adult populations would reduce the need for 22-42 million treatments, resulting in over 3 billion US dollars saved over a 20 yr deployment period.^6^ Importantly, TB vaccines are also ranked as having elevated feasibility for reducing antimicrobial resistance,^7^ an alarming problem across bacterial pathogens. In 2023 alone, the WHO estimates there were 400,000 cases of rifampicin resistant or multidrug resistant M.tb, with a pitiful 68% treatment success rate.^3^ Given their promise, we and others in the field are working to backfill the preclinical pipeline with novel TB vaccine candidates. Presently, there are only 13 vaccine candidates against TB under active clinical development;^7^ this is in stark contrast to the 50 approved vaccines, 242 candidates, and 821 vaccine trials for severe acute respiratory syndrome coronavirus 2 (SARS-CoV-2) reported by the WHO COVID19 Vaccine Tracker by the end of 2022.

Vaccine candidates against infectious pathogens are developed in many platforms, including subunit vaccines which combine proteins with formulated adjuvants.^8^^,^^9^ Adjuvants are critical for driving potent immune responses against important pathogen-derived proteins, traditionally not highly immunogenic, by taking advantage of key pattern recognition receptors (PRR) and Toll-like receptor (TLR) pathways.^10–12^ Adjuvants can also play an important role in overcoming immune senescence brought on by aging, where the immune response wanes in magnitude and composition.^13^^,^^14^ Antigen selection and choice of adjuvant have thus far driven the preclinical and early clinical successes of current subunit TB vaccine candidates. Clear immune correlates make adjuvant selection simpler and rational in their approach. In the absence of correlates, as is the case with protection against TB,^15–17^ several strategies have been leveraged to date that target TLR receptor signaling, including TLR4 and TLR9 agonists which help drive canonical T-helper 1 (T_H_1) biased immunity and proinflammatory cytokine responses like interferon gamma (IFN-γ).^18^^,^^19^ There has been a recent call to diversify the availability of candidate antigens and adjuvants.^15^^,^  ^20–22^ We hypothesize that novel TLR4 adjuvants may provide depth of availability in cases of limited resources.

Here in this work, we executed a suite of escalating preclinical experiments to evaluate a novel adjuvant leveraging synthetic monophosphoryl lipid A (SyMPL), a detoxified derivative of lipopolysaccharide which activates TLR4,^23^ formulated as an oil-in-water emulsion (Emulsion TLR4 agonist [EmT4™]). The natural analog, MPL, has been used successfully in both prophylactic and therapeutic vaccines.^24–26^ Synthetic TLR4 based agonistic adjuvants can be tailored for improved immune responses, for example glucopyranosyl lipid adjuvant (GLA) compared with second generation lipid adjuvant (SLA).^27^ In addition, whereas HPLC shows the presence of a heterogeneous composition of MPL molecular species, derived from bacterial preparations, the synthetic agonists are more defined and therefore may be more amenable to rigorous quality control and technology transfer to other sites around the globe—ideal for modern manufacturing and global vaccine applications.^23^^,^  ^28^^,^^29^ One of the most advanced TLR4 agonist based adjuvants, adjuvant system 01 (AS01), contains MPL and QS21, and is dependent on a natural source of from the Auillaja Saponaria soap bark tree (*Quillaja saponaria*). This dependence becomes resource limiting and less sustainable;^30^ therefore, synthetic versions are currently being considered and pursued.^31^ In these studies, we chose SyMPL as the TLR4 agonist in an emulsion to develop immunogenic and protective TB vaccine candidates.^32–34^ We observed that formulated EmT4™ activates mouse-derived macrophages and dendritic cells *ex vivo*, is well tolerated and induces humoral and cellular immunity when administered intramuscularly with antigen. We used clinical TB vaccine candidate antigen ID93^33^^,^  ^35–37^ as well as next generation ID91 protein fusions^38^ as representative candidates to formulate with EMT4™. Excitingly, this resulting immune response confers *in vivo* prophylactic protection from aerosol challenge to two separate M.tb strains across models of young and aged mice.

## Materials and methods

### Preclinical animal model

Female C57BL/6 mice 4-6 weeks of age were purchased from Charles River Laboratory (Wilmington, Massachusetts, USA). Additional male C57BL/6 bg/bg (Beige) mice were bred in-house (Original source Jackson Laboratory: C57BL/6J-Lystbg-J/J, Bar Harbor, Maine, USA) and used for experiments at 6 to 8 wk (young) or 20 mo (aged). Mice were housed at the Seattle Children’s Research Institute (SCRI) biosafety level 3 (BSL-3) animal facility under pathogen-free conditions and were handled in accordance with experimental protocols that were approved by the SCRI Institutional Animal Care and Use Committee (IACUC). All methods were carried out in accordance with relevant guidelines and regulations with respect to animal welfare. Across experiments mice were co-housed by cohort throughout the study timeline. Mice were infected with a low dose (50 to 100 bacteria) aerosol (LDA) of M.tb HN878 (BEI resources) or M.tb H37Rv (ATCC No. 35718; American Type Culture Collection, Manassas, Virginia, USA) using a Glas-Col aerosol infection chamber (Glas-Col, LLC, Terre Haute, Indiana, USA). Twenty-four hours post challenge the lungs of 3 mice were homogenized and plated on Middlebrook 7H10 agar (Fisher Scientific, Waltham, Massachusetts, USA) to confirm bacterial delivery.

### M.tb fusion antigens and EmT4™ adjuvant

The ID93 antigen is a recombinant fusion of the M.tb proteins Rv2608, Rv3620, Rv1813, and Rv3619.^38^ The ID91 antigen is a recombinant fusion of the M.tb proteins Rv3619, Rv2389, Rv3478, and Rv1886.^38^ Both ID93 and ID91 have been shown to induce prophylactic protection in a C57BL/6 mouse model of TB when partnered with adjuvants.^39–45^

EmT4™ is a novel adjuvant produced by PAI Life Sciences Inc. (Seattle, WA) containing potent TLR4 agonist, 3D (6-acyl)-PHAD (Croda International, Snaith, United Kingdom). The TLR4 agonist is formulated in an oil-in-water (o/w) emulsion consisting of metabolizable oils emulsified with biocompatible surfactants in an aqueous bulk phase. Emulsion droplets are ∼100 nm in diameter and comparable formulations have proven to be stable for years, stability studies of EmT4™ itself are ongoing. As an amphiphilic molecule, TLR4 agonists are thought to localize at the oil/water interface.

### Tolerability

FDA-like requirements for tolerability were used to evaluate EmT4™ including: max number of doses +1 (4 doses total), target dose (5 μg), target dose 2× (10 μg), and target dose 3× (15 μg). C57BL/6 female mice were weighed daily during this tolerability study, and were examined visually for overt signs of morbidity including ruffled fur, lack of movement, huddled, or hunched posture. At the end of the study (D12) plasma ALT, AST liver enzyme levels, C-reactive protein (CRP) were evaluated in mouse-specific commercial ELISAs (Abcam), and whole blood was analyzed for whole blood cell, red blood cell, and platelet counts using a DxH cell analyzer (Beckman Coulter). Individual organs including lung, spleen, kidneys, and liver were weighed at study termination.

### Immunizations

Cohorts of mice were immunized intramuscularly (i.m.) 3 times 3 weeks apart, with the final immunization occurring 4 weeks before challenge with M.tb HN878 or M.tb H37Rv. Female C57BL/6 mice were immunized with saline, 5 μg EmT4™ alone, or 0.5 μg of ID93 combined with 5 μg of EmT4™ where a total immunization volume of 100 µl was spread across both hind limbs. Control mice were immunized once intradermally (i.d.) with 50 μl at the base of the tail containing 10^4^ CFU of bacillus Calmette–Guérin (BCG) (Pasteur strain, AERAS) 10 weeks before challenge, and at the same time as the protein-adjuvant prime for other cohorts. Separate cohorts of young (6 to 8 wk) and aged (20 mo) male Beige mice were immunized i.m. with saline or 0.5 μg of ID91 combined with 5 μg of EmT4™ 2 times 3 weeks apart. Mice were then challenged with an LDA aerosol of M.tb (either M.tb H37Rv or M.tb HN878, as described above) 3 weeks after the boost immunization.

### Bone marrow-derived dendritic cells (BMDC)

BMDC were culture-induced *in vitro* using sterile aspirated bone marrow from the femurs of C57BL/6 mice, as previously published.^46^ Briefly, bone marrow cell suspensions were prepared at a concentration of 10^6^ cells/ml in complete media (RPMI 1640, 10% fetal bovine serum, 1% L-glutamine, 1% penicillin/streptomycin) supplemented with 20 ng/ml recombinant mouse GM-CSF and 20 ng/ml IL-4 (PeproTech, Rocky Hill, New Jersey, USA). On day 3, DCs were supplemented with complete RPMI media and 20 ng/ml GM-CSF. On day 7, cells were collected with moderately vigorous pipetting, counted and plated at 2 × 10^5^ cells/well in fresh complete RPMI media and stimulated for 48 h at 37°C with 0.1 to 100 ng/ml of EmT4™, LPS (Millipore Sigma, Burlington, Massachusetts, USA) or equivalent volume of PBS. After 48 h cells were surface stained for 20 min at room temperature with the following antibodies: CD11c (Bv650, clone N418, Biolegend, San Diego, California, USA), CD86 (APC, clone GL-1, Biolegend), CD40 (PE/Cy7, clone 3/23, Biolegend), and MHCII I-A/I-E (eF450, clone M5/114.15.2, Invitrogen, Waltham, Massachusetts, USA). Samples were acquired on an LSRII flow cytometer (BD Biosciences, Franklin Lakes, New Jersey, USA) with FACSDiva software (BD Biosciences). Gating strategy is shown in Fig. S1A, and analysis was performed using FlowJo v10.6.1 (BD Biosciences).

### Bone marrow-derived macrophages (BMDM)

BMDM from C57BL/6 mice were culture-induced *in vitro* as previously published^47^ with modifications as described. Bone marrow cells were suspended at a concentration of 10^6^ cells/ml in complete medium (DMEM, 10% fetal bovine serum, 1% penicillin/streptomycin) supplemented with 10 ng/mL recombinant mouse M-CSF1 (PMC2044, Gibco, Grans Island, New York, USA). On day 3, macrophages were supplemented with complete DMEM media and 10 ng/mL M-CSF1. On day 7, cells were collected with moderately vigorous pipetting, counted and were plated at 2 × 10^5^ cells/well in fresh complete DMEM medium and stimulated for 48 h at 37°C with 0.1 to 100 ng/ml of EmT4™, LPS, or PBS. After 48 h cells were surface stained for 20 min at room temperature with the following antibodies: CD11b (PE, clone M1/70, BD Biosciences), CD86 (APC, clone GL-1, Biolegend), CD40 (PE/Cy7, clone 3/23, Biolegend), and MHCII I-A/I-E (eF450, clone M5/114.15.2, Invitrogen). Samples were acquired on a LSRII flow cytometer (BD Biosciences) with FACSDiva software (BD Biosciences). Gating strategy is shown in Fig. S1B, and analysis was performed using FlowJo v10.6.1 (BD Biosciences).

### Multiplex cytokine analysis

Serum samples were isolated using 1.1 ml serum gel tubes (SAI Infusion technology, Lake Villa, Illinois, USA) and whole blood terminal bleeds immediately following euthanasia 4 wk after the third immunization and 4 wk after infection with M.tb HN878 or M.tb H37Rv. Circulating serum cytokines were measured using the Meso Scale Discovery (MSD) mouse V-PLEX Plus Mouse Cytokine 19-Plex Kit (Meso Scale Discovery, Rockville, Maryland, USA) that included the following analytes: IFN-γ, IL-1β, IL-2, IL-4, IL-5, IL-6, IL-9, IL-10, IL-12p70, IL-15, IL-17A/F, IL-27p28/IL-30, IL-33, IP-10, KC/GRO, MCP-1, MIP-1α, MIP-2, and TNF-α. Serum was added in a 1:2 dilution to plates pre-coated with cytokine-specific antibodies following manufacturer’s instructions. Concentration of each cytokine were determined using the MSD Discovery Workbench analysis software (Meso Scale Discovery, Rockville, Maryland, USA). Values below the limit of detection of the assay were assigned a value half that of the lowest standard. The levels of IL-9 and IL-4 were below the limit of detection for all animals at both time points. The results are reported as the means from duplicate wells in pg/ml.

### Antibody ELISAs

Serum samples were initially diluted 1:100 and added to the first well of 384-well plates and then serially diluted onto plates coated with 2 µg/ml of ID93 or ID91 aligned with experimental design and blocked as previously described.^48^ After an overnight incubation, plates were exposed to HRP-conjugated Total IgG, IgG1, or IgG2c antibodies (Southern Biotech, Birmingham, Alabama, USA). Plates were then developed with tetramethylbenzidine (TMB) substrate (KPL/Seracare, Milford, Massachusetts, USA) and stopped with 1 N H_2_SO_4_ (Fisher Scientific). Plates were read on a SpectraMax iD3 microplate reader (Molecular Devices, San Jose, California, USA) at 450 nm with 570 nm background subtraction. Reciprocal dilutions corresponding to endpoint titers (EPT) were determined with GraphPad Prism 9.4.1 (GraphPad Software, San Diego, California, USA) with a cutoff value determined by the average of saline treated serum control wells + 2 times the standard deviation.

### Flow cytometry

Intracellular flow cytometry (ICS) was performed on cells from mouse spleen or lung homogenates 4 wk after the third immunization and 4 wk after infection with M.tb HN878 or M.tb H37Rv, respectively. Samples were washed and stimulated as previously described.^48^ Briefly, cells were stimulated with media alone, 10 µg/ml of recombinant ID93, or 1 µg/ml phorbol myristate acetate (PMA) (Calbiochem, San Diego, California, USA) + 1 µg/ml ionomycin (Sigma-Aldrich, St Louis, Missouri, USA), and incubated at 37°C. After 2 h of incubation, 1 µg/µl of GolgiPlug (BD Biosciences) was added and samples were incubated at 37°C for an additional 10 h. Samples were held at 4°C until staining. After stimulation, samples were stained for markers of interest using fluorophore conjugated antibodies. Primary surface staining included anti-mouse CD4 (clone RM4-5, BioLegend), CD8 (clone 53-6.7, BioLegend), CD44 (clone IM7, eBioscience, San Diego, California, USA), and 1 µg/ml of Fc receptor block anti-CD16/CD32 (clone 93, eBioscience) in PBS with 1% bovine serum albumin (BSA, Sigma). Cells were then washed with PBS/BSA and fixed using BD Biosciences Fix/Perm reagent for 20 min at RT then washed with Perm/Wash buffer (BD Biosciences). Intracellular staining was performed in Perm/Wash with anti-mouse IFN-γ (clone XMG1.2, Invitrogen), IL-2 (clone JES6-5H4, eBioscience), TNF-α (clone MP6-XT22, eBioscience), IL-5 (clone TRFK5, BioLegend), IL-17A (clone TC11-18H10.1, BioLegend), GM-CSF (clone MP1-22E9, BioLegend), and CD154 (clone MR7, eBioscience) for 15 min at RT. All antibodies were used at 1:100 dilution from the stock vial. Samples were acquired on a LSRII flow cytometer (BD Biosciences) with FACSDiva software (BD Biosciences). Gating strategies were based on our previous publications^48–50^ (and shown in Fig. S1C), and analysis was performed using FlowJo v10.6.1 (BD Biosciences) and SPICE (National Institutes of Health, http://exon.niaid.nih.gov/spice) reported values are background (unstimulated controls) subtracted. Fluorescence Minus One (FMO) controls were used to accurately define gating thresholds by omitting one fluorophore at a time, while non-stained cells served as a baseline for fluorescence. Fully stained cells were used to evaluate the staining profile and confirm marker identification (Fig. S2).

### Bacterial burden

At 24 h and 4 wk post infection with M.tb HN878 or M.tb H37Rv, 3 mice total or 7 mice per group, respectively, were euthanized, and bacterial counts were enumerated as described previously.^48–50^ Briefly, mice were euthanized with CO_2_, and lung and spleen tissues from infected animals were isolated and homogenized in 2 ml (24 h) or 5 ml (4 wk) of either RPMI + FBS (lung) or PBS + 10% Tween-80 (spleen) (Sigma-Aldrich, St Louis, Missouri, USA) using an Omni tissue homogenizer (Omni International, Kennesaw, Georgia, USA). For 24 h samples, total lung homogenates were plated neat across four Mitchison 7H11 selective agar monoplates (containing carbenicillin, polymyxin B, amphotericin B and trimethoprim, Fisher Scientific). For week 4 samples, serial dilutions of homogenate were prepared in PBS + 10% Tween-80 and aliquots were plated on Middlebrook 7H10 agar tri-plates supplemented with OADC enrichment by the supplier (Molecular Toxicology, Boone, NC) and subsequently incubated at 37°C for approximately 3 wk before colonies were counted. Bacterial burden, indicated as colony forming units/mL (CFU/mL), was calculated per organ and is presented as Log_10_ values. Reduction in the bacterial burden was calculated as the difference in mean Log_10_ values between the groups.

### Statistical analysis

Weights over time between cohorts in the tolerability study were compared using a two-way ANOVA with Dunnett’s multiple comparison test. Circulating cytokine levels and humoral immune responses at both post-immunization and post M.tb HN878 and M.tb H37Rv infection timepoints were compared between vaccinated groups and saline control using 2-way ANOVA with Tukey’s multiple comparisons test. Bacterial burden and magnitude of polyfunctional T cell responses at a single timepoint were assessed using a 1-way ANOVA with Dunnett’s multiple comparisons test between vaccinated groups and saline control with subtraction of medium background. All the above analyses were performed using GraphPad Prism 9.4.1. Significant differences are labeled accordingly in the figures where *P < 0.05, **P < 0.01, ***P < 0.001, and ****P < 0.0001, as previously described by our group.^48–50^

## Results

### Formulation of EmT4™ adjuvant

**Figure 1.**
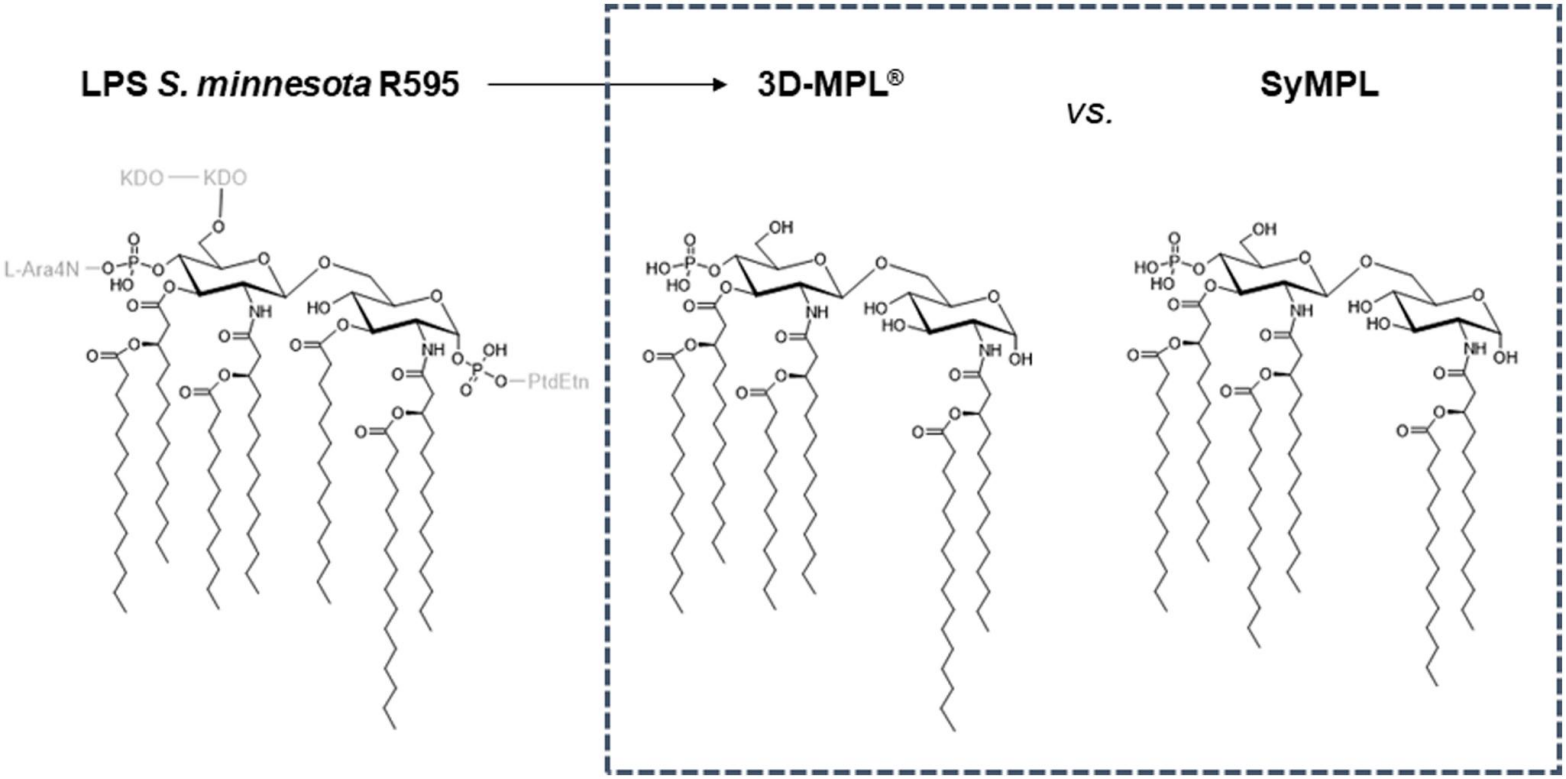
Comparison of natural core structure of LPS with SyMPL. Extensive similarity between MPL derived from naturally occurring LPS (left) and the synthetic TLR4 ligand (SyMLP, right) can be seen (box). To produce 3D-MPL^®^ (center), LPS from S. Minnesota R595 (left) is treated with acid and base hydrolyzing some of the ester bonds and leading to a mixture of multiple acyl-species of MPL, the active form of which is shown above. *Note*. L-Ara4N = 4-amino-4-deoxy-L-arabinose; PtdEtn = phosphatidylethanolamine; KDO = 3-deoxy-D-manno-octulosonic acid.

The 3D (6-acyl)-PHAD is a synthetic compound, SyMPL (Fig. 1), which acts as a potent stimulator of TLR4 and is similar in structure and function to the active ingredient in adjuvants like GSK’s AS0x series of adjuvants included in the approved cervical cancer vaccine Cervarix^®^ and the shingles vaccine Shingrix^®^. The molecule is released using NMR data indicating it does not have multiple species post synthesis (data not shown). The emulsions pH is chosen to not hydrolyze the ester bonds connecting the acyl chains to the sugar core so in the emulsion the active agonist retains its structure.

### Dose-dependent BMDC and BMDM responses to EmT4™ adjuvant

**Figure 2.**
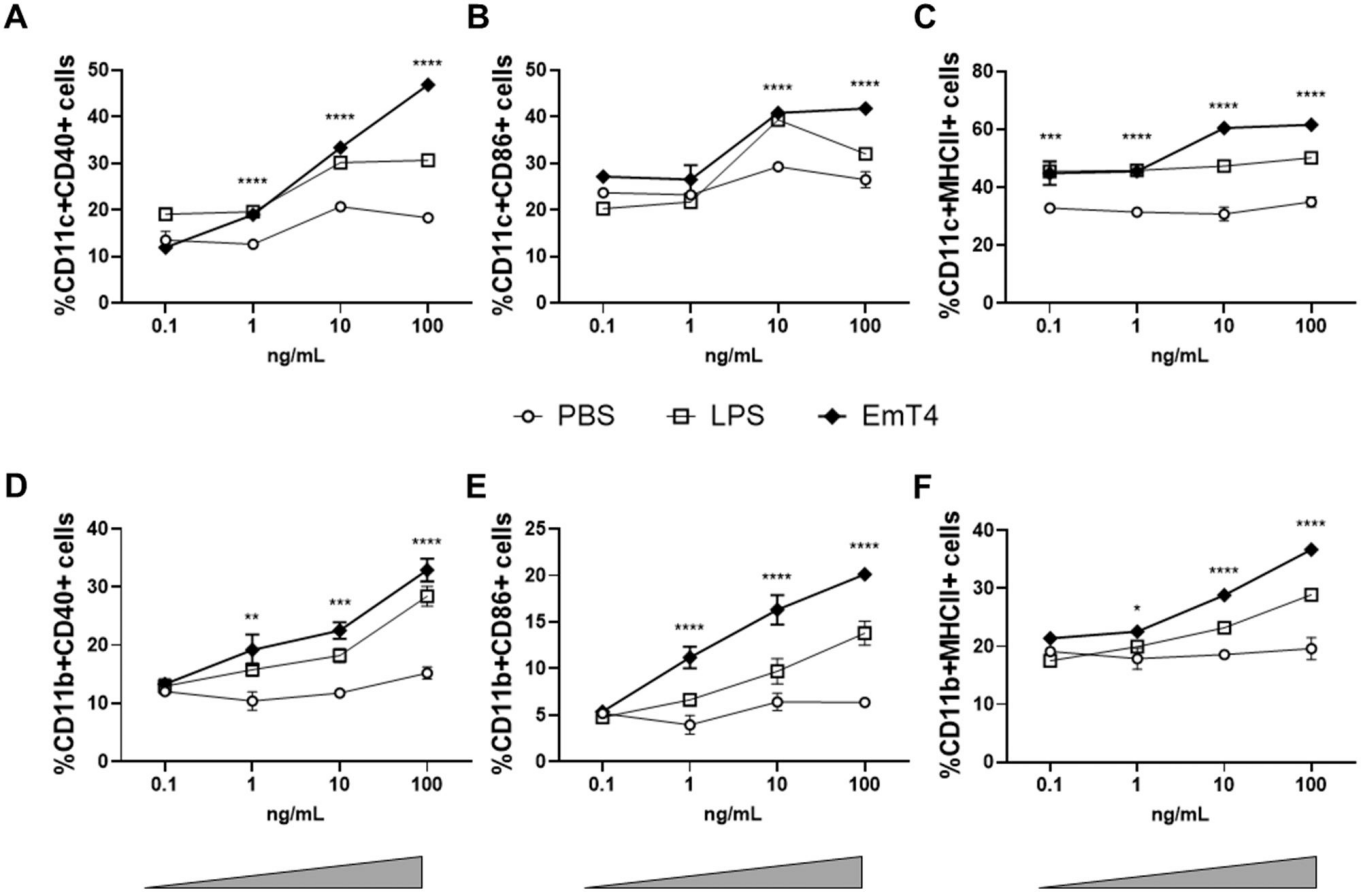
EmT4™ adjuvant activates innate immune cells and enhances antigen presentation. Activation of bone marrow derived dendritic cells (BMDC) and macrophages (BMDM) with EmT4™ adjuvant. BMDC (A–C) and BMDM (D–F) from naïve C57BL/6 mice were stimulated with increasing concentrations (0.1–100 ng/mL) of EmT4™ (closed diamonds), LPS (open circle) or PBS (open square), and surface stained for activation markers after 48 h. CD11c+ BMDCs and CD11b+ BMDMs were evaluated for the percentage of cells co-expressing CD40 (A, D), CD86 (B, E) and MHCII (C, F). Data shown are mean ± SEM from n = 5 technical replicates per treatment, representative of 2 experiments. Asterisks indicate statistical significance of EMT4™ treatment compared to the PBS group at each dose, where *P < 0.05, **P < 0.01, ***P < 0.001, and ****P < 0.0001 using two-way ANOVA with Šídák's multiple comparisons test.

Adjuvants activate innate immunity by inducing cytokine and chemokine expression in antigen presenting cells (APC), including dendritic cells (DC) and macrophages, and participate in the maturation of these cells.^51–53^ A hallmark of DC and macrophage activation is the upregulation of co-stimulatory molecules on the cell surface and increased antigen presentation capability. Therefore, we first investigated surface expression of activation and costimulatory markers on naïve mouse bone marrow-derived dendritic cells (BMDC) and macrophages (BMDM) across a dose curve of EmT4™ stimulation. EmT4™ induced a dose-dependent increase in CD40, CD86, and MHC class II activation markers on the cell surface of both CD11c^+^ DC (Fig. 2A–C) and CD11b^+^ macrophages (Fig. 2D–F). These data indicate that EmT4™ adjuvant productively activates DCs and macrophages in a dose-dependent manner and may enhance the capacity for antigen presentation. As a benchmark we used LPS which potently activated these cells.

### Maximum tolerated dose

**Figure 3.**
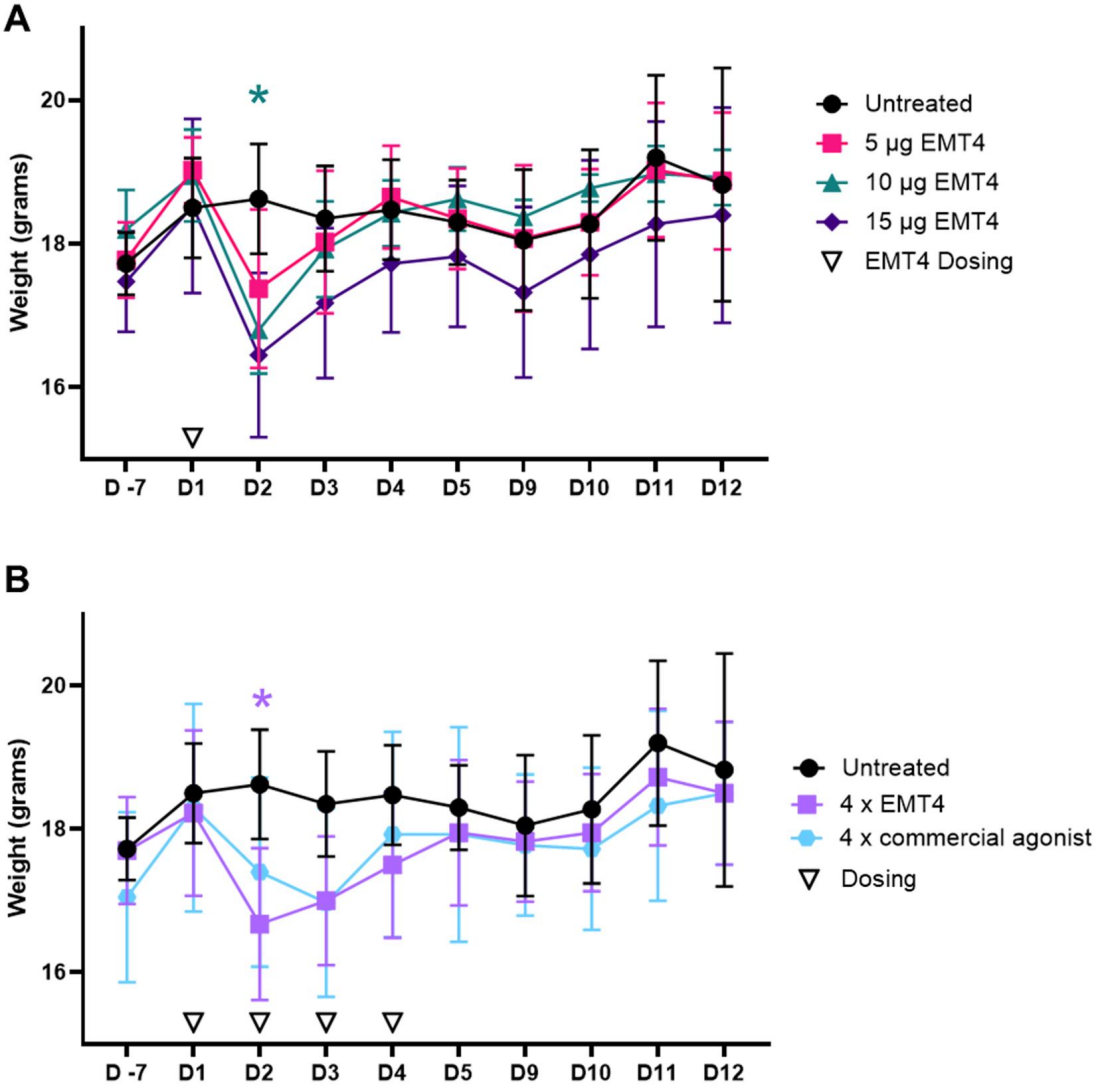
Maximum tolerated dose study. (A) C57BL/6 mice were given a single immunization with 5 µg (pink squares), 10 µg (teal triangles), or 15 µg (purple diamonds) of EMT4™ and weighed through day 12 along with untreated cohorts (black circles). (B) In a separate study cohorts were given 4 consecutive immunizations with 5 µg of EMT4™ (purple squares), or 5 µg of a commercial TLR4 agonist (blue hexagons) and weighed through day 12 along with untreated cohorts (black circles). Dosing intervals are denoted by open upside down triangles along the *X*-axis. Treated cohorts were individually compared to untreated using a 2-way ANOVA with Dunnetts multiple comparisons, significant differences are denoted where *P < 0.05. Data representative of one experiment with n = 4 animals per group.

Given this is a novel adjuvant formulation we assessed EmT4™ using FDA-like requirements for tolerability including max number of doses +1 (4 doses total), target dose (5 ug), target dose 2× (10 ug) and target dose 3× (15 ug). Our primary readout for tolerability included whole body weights over time. When comparing weights of cohorts that received a single immunization of escalating doses, only 10 µg of EMT4™ was significantly lower (*P* = 0.0251) than untreated animals on day 2 post treatment (Fig. 3A). All single doses demonstrated an expected dip in weight after immunization, but all cohorts recovered by day 5 (Fig. 3A) as we have previously observed in vaccine studies. When giving repeated treatments for 4 consecutive days we observed that the EMT4™ (5 ug) cohort was again significantly lower (*P* = 0.0476) than untreated animals by day 2, but recovery was noted by day 5 (Fig. 3B). We included a commercially available TRL4 agonist as a control in our repeated dosing study and observed no major differences in weights over time when compared to EMT4™ treated cohorts (Fig. 3B). There was no notable trend nor statistical difference in lung, kidney, spleen, or liver organ weights at day 12 post study start between any cohort in the tolerability study (data not shown). At day 12 post study start, whole blood was collected for evaluation on a DxH 500 cell analyzer and for plasma isolation. Using plasma, we evaluated alanine aminotransferase (ALT) and aspartate aminotransferase (AST) liver enzyme levels as markers of liver stress, and circulating levels of C-reactive protein (CRP) as a measure of overt inflammation (Fig. S3A–C) and found no differences between any treatment groups. Whole blood analysis similarly revealed little differences between groups in the whole blood cell count (Fig. S3D), red blood cell count (Fig. S3E), or platelet count (Fig. S3F). We did observe a small but significant increase in the number of whole blood cells in the group receiving 3× target dose (15 µg EMT4™) compared to the untreated control (Fig. S3D). Given EMT4™ was well tolerated alone, we next evaluated the immunogenicity and efficacy against M.tb challenge in the mouse model when paired with priority TB vaccine candidates.

### Prophylactic immunogenicity and protective efficacy

#### Study design

**Figure 4.**
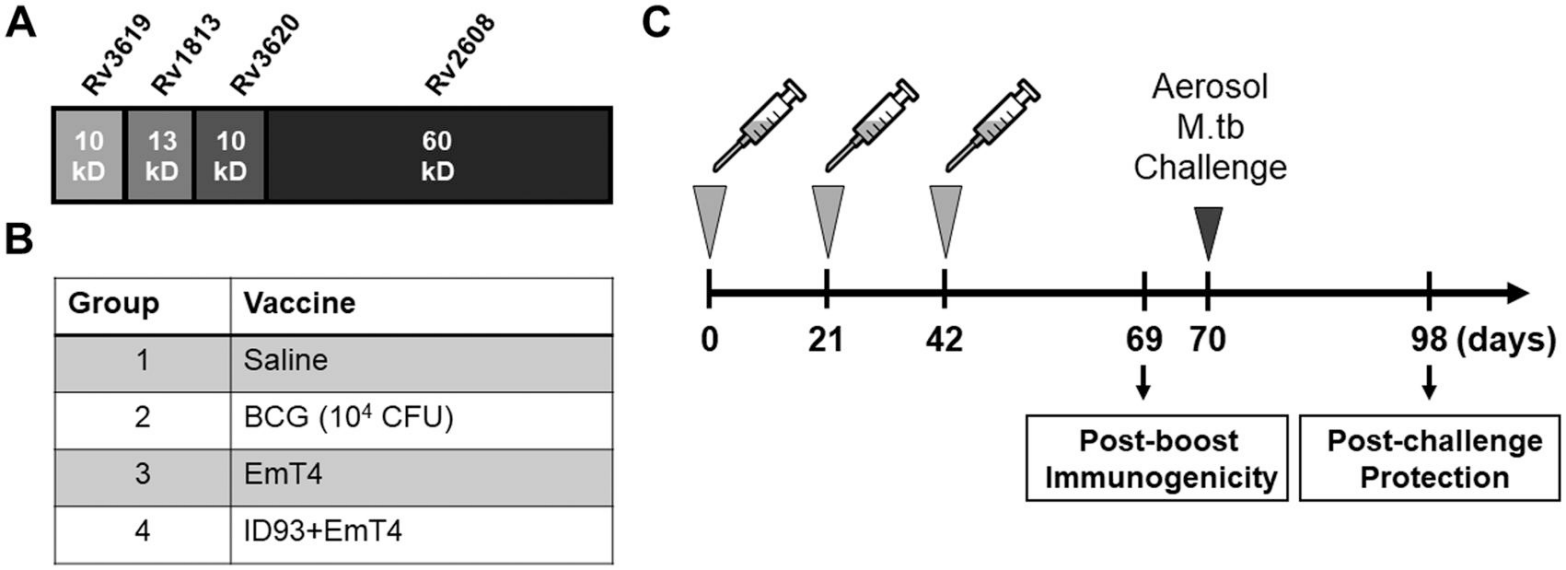
Overview of experimental design and immunizations. (A) Schematic of the ID93 fusion antigen comprising 4 M.tb proteins with the molecular weight of each component. (B) Cohort design of vaccine regimen per group. (C) Timeline of immunizations and aerosol M.tb HN878 and M.tb H37Rv challenge. Experimental samples were collected at 2 time points: D69 (4 wk post-third immunization) and D98 (4 wk post- M.tb challenge). Immunogenicity timepoints included n = 4 mice per group whereas protection endpoints included n = 7 per group. Experiment performed one time for each M.tb challenge.

In this study we combined the EmT4™ adjuvant with clinical vaccine candidate, ID93 (Fig. 4A), to assess the prophylactic efficacy against two strains of M.tb aerosol challenge in a mouse model. Female C57BL/6 mice were immunized with either saline, BCG, EmT4™ alone, or ID93+EmT4™ 3 times, 3 wk apart (Fig. 4B, C). Vaccine-induced preliminary immunogenicity was assessed 4 wk post- the third immunization. Mice were then aerosol challenged with M.tb HN878 or M.tb H37Rv and vaccine-induced protection was assessed four weeks post-infection (Fig. 4C). We selected M.tb HN878 as a representative lineage 2 Beijing clinical isolate and M.tb H37Rv as a representative lineage 4 laboratory strain, both of which are widely used by our group and others.

#### Systemic proinflammatory response

**Figure 5.**
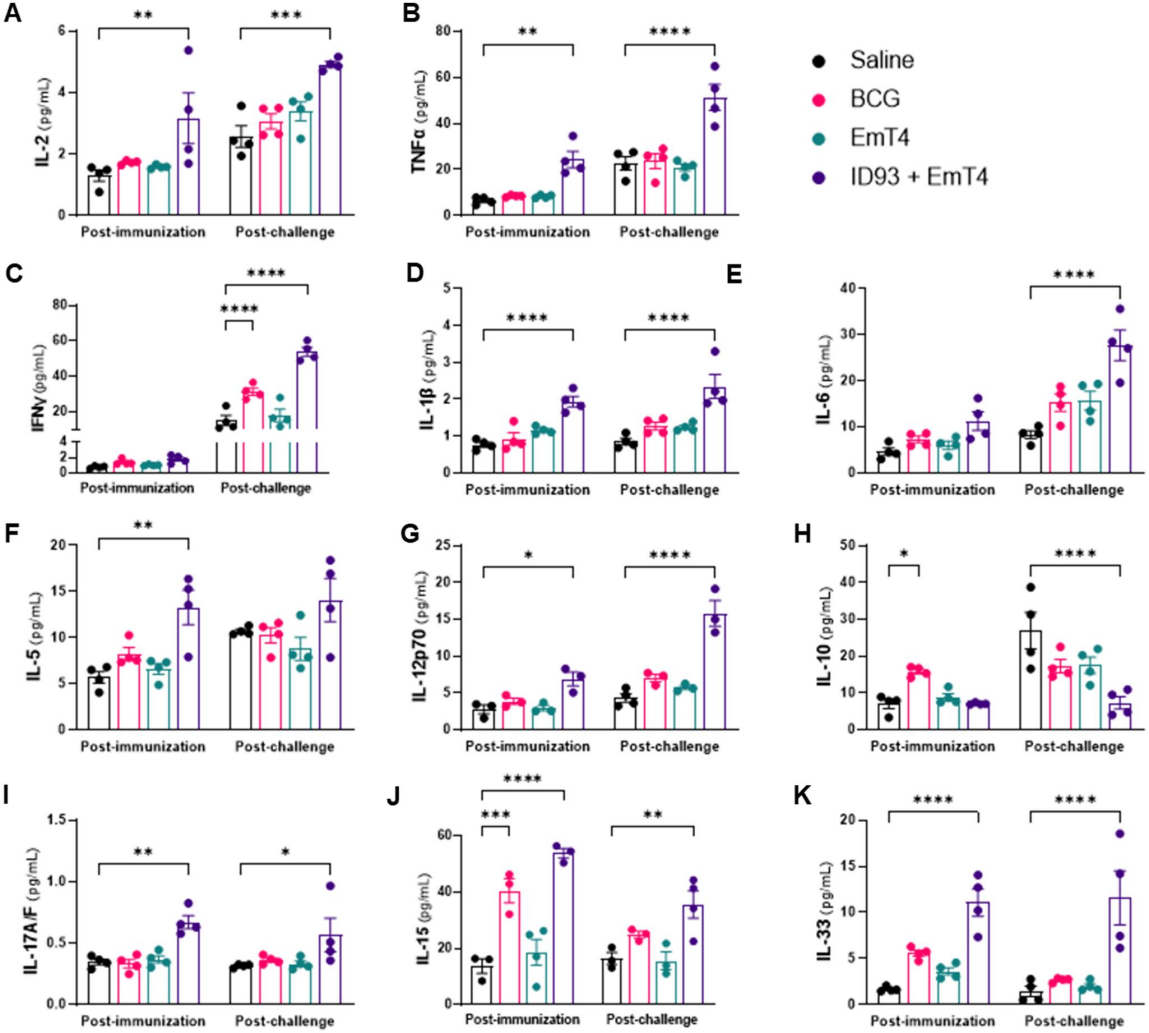
Vaccination with ID93+EmT4™ induces robust circulating cytokine responses post-immunization and is still detectable post-M.tb HN878 challenge. Systemic cytokine levels were measured in serum samples collected 4 wk after the third immunization and 4 wk after challenge with M.tb HN878. Concentration of (A) IL-2, (B) TNF-α, (C) IFN-γ, (D) IL-1β, (E) IL-6, (F) IL-5, (G) IL-12p70, (H) IL-10, (I) IL-17A/F, (J) IL-15, and (K) IL-33 pg/mL post-immunization (left) and post-challenge (right). Bars show mean ± SEM, dots represent individual mice, n = 3-4/group. Asterisks indicate statistical significance compared to the saline group, where *P < 0.05, **P < 0.01, ***P < 0.001, and ****P < 0.0001 using 2-way ANOVA with Tukey’s multiple comparisons test.

Challenge with M.tb leads to infection of macrophages, monocytes, and dendritic cells and a subsequent decrease in TNF-α, IFN-γ, and other proinflammatory cytokines.^54–57^ Conversely, there is an increase in IL-10 production which promotes intracellular M.tb survival within the host. Therefore, we evaluated vaccine-induced circulating IL-2, TNF-α, IFN-γ, IL-1β, IL-6, IL-5, IL-12p70, IL-10, IL-17A/F, IL-15, and IL-33 levels in serum samples collected 4-week post third immunization and 4-weeks post M.tb HN878 (Fig. 5A–K) or M.tb H37Rv challenge (Fig. S4A–K). Four weeks after the third immunization, we observed a significant increase in circulating TNF-α, IL1-β, IL-2, IL-5, IL-12p70, IL17A/F, IL-15 and IL-33 levels in C57BL/6 mice immunized with ID93+EmT4™ compared to saline controls. Interestingly, there is a significant increase in circulating IL-10 and IL-15 levels in the serum of the BCG immunized group (Fig. 5H). Four weeks post infection with M.tb HN878, we observed a significant increase in circulating IFN-γ, TNF-α, IL-1β, IL-2, IL-6, IL-12p70, IL-17A/F, IL-15, and IL-33 levels in mice immunized with ID93+EmT4™ compared to saline controls. We also observed a significant increase in circulating IFN-γ levels in the serum of the BCG immunized group after infection with M.tb HN878 (Fig. 5A). Lastly, there was a significant decrease in circulating IL-10 levels post M.tb HN878 infection in serum of the ID93+EmT4™ immunized group (Fig. 5H). These data indicate that immunization with ID93+EmT4™ elicits a strong proinflammatory response that is detectable systemically both post-immunization and post-infection with either M.tb HN878 (Fig. 5) or M.tb H37Rv (Fig. S4).

#### Circulating chemokine response

**Figure 6.**
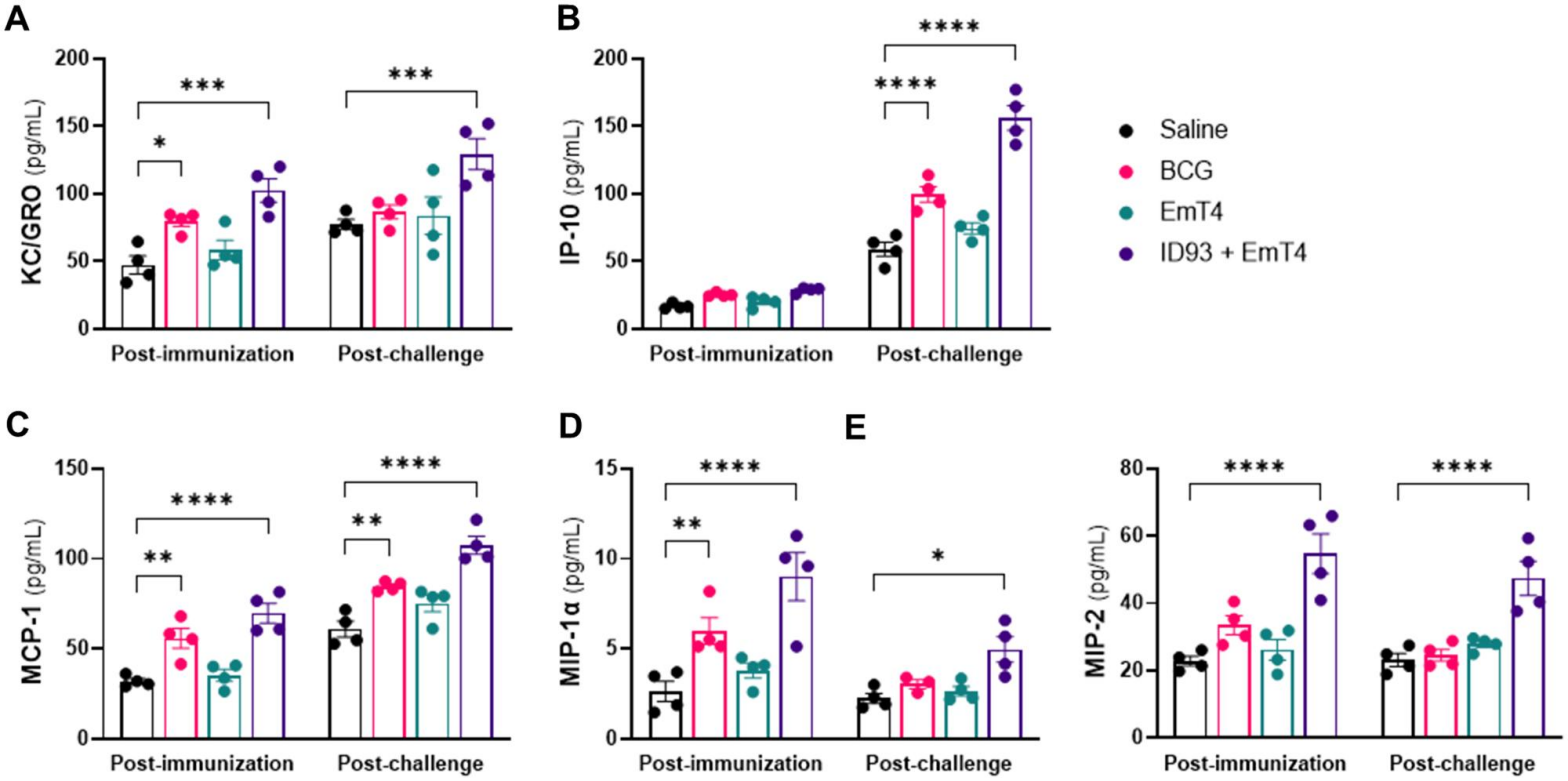
Vaccination with ID93+EmT4™ induces strong circulating chemokine responses post-immunization and is detectable post-M.tb HN878 challenge. Circulating chemokine levels were measured in serum samples collected 4 wk after the third immunization and 4 wk after challenge with M.tb HN878. Concentration of (A) KC/GRO, (B) IP-10, (C) MCP-1, (D) MIP-1α, and (E) MIP-2 pg/mL post boost immunization (left) and post challenge (right). Bars show mean ± SEM, dots represent individual mice, n = 4/group. Asterisks indicate statistical significance compared to the saline group, where *P < 0.05, **P < 0.01, ***P < 0.001, and ****P < 0.0001 using two-way ANOVA with Tukey’s multiple comparisons test.

Chemokines have been shown to control cell influx to M.tb-infected lungs and play a critical role in TB infection control.^58^^,^^59^ Therefore, we also evaluated vaccine-induced circulating KC/GRO, IP-10, MCP-1, MIP-1α, and MIP-2 levels in serum samples collected 4-week post- third immunization and 4-weeks post-M.tb HN878 (Fig. 6A–E) or M.tb H37Rv challenge (Fig. S4L–P). Four weeks after the third immunization, we observed a significant increase in KC/GRO, MCP-1, MIP-1α, and MIP-2 levels in mice immunized with ID93+EmT4™. We also observed a significant increase in circulating KC/GRO, MCP-1, and MIP-1α levels in the serum of the BCG immunized group. Four weeks post-infection with M.tb HN878, we observed a significant increase in circulating KC/GRO, IP-10, MCP-1, MIP-1α, and MIP-2 levels in mice immunized with ID93+EmT4™. We also observed a significant increase in circulating IP-10 and MCP-1 levels in the serum of the BCG immunized group after infection with M.tb HN878. These data indicate that immunization with ID93+EmT4™ elicits a robust *ex vivo* circulating chemokine response both post-immunization and post-infection with M.tb HN878 and M.tb H37Rv.

#### Humoral and cellular adaptive immune response

**Figure 7.**
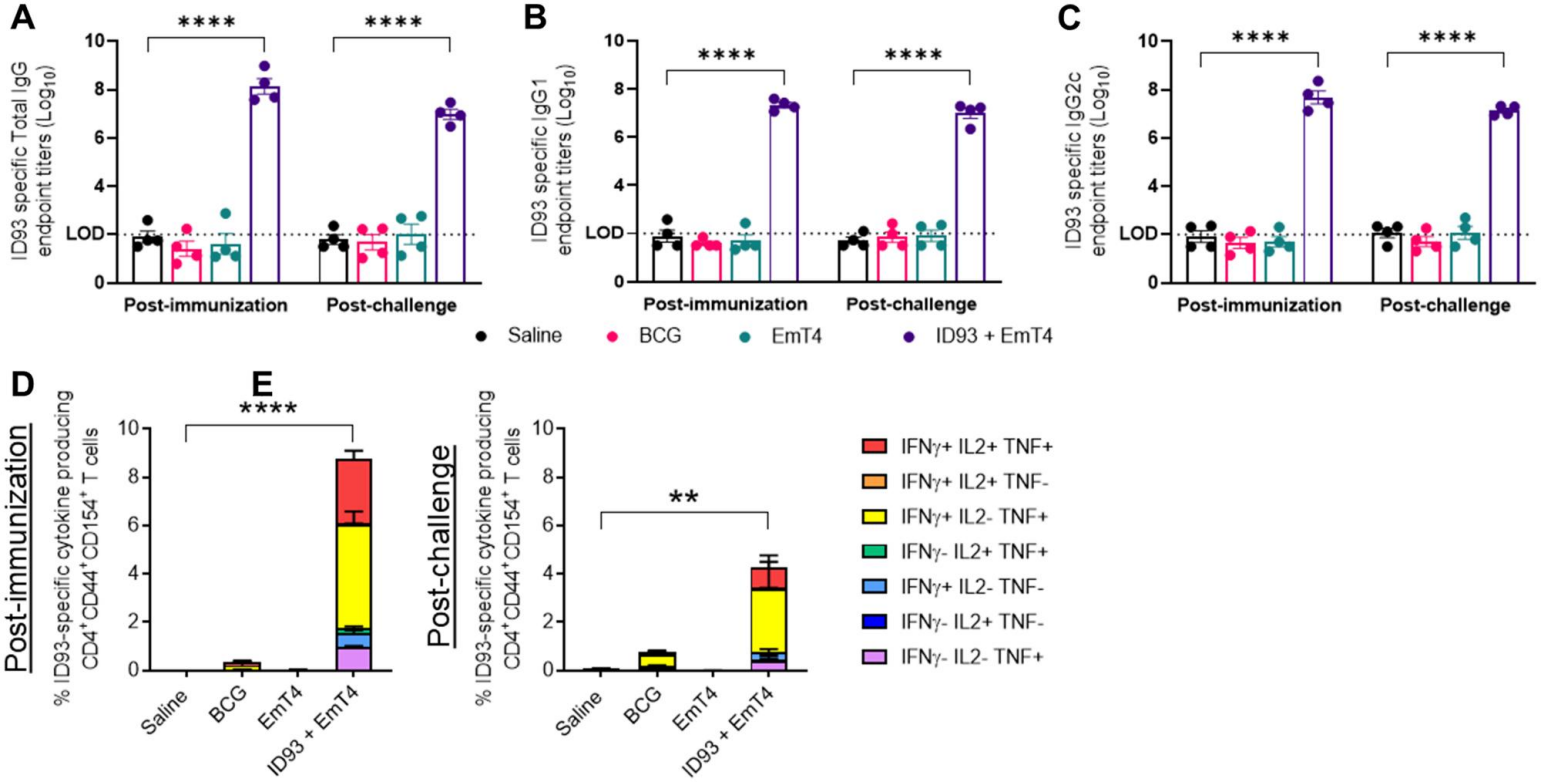
Vaccination with ID93+EmT4™ drives ID93 antigen-specific humoral and cellular immune responses post-immunization and post-M.tb HN878 challenge. Serum samples were collected 4 wk after the third immunization and 4 wk after infection with M.tb HN878 and were evaluated for: ID93 antigen-specific (A) Total IgG, (B) IgG1, (C) and IgG2c responses. Log_10_ endpoint titer (EPT) is shown (LOD = limit of detection of the assay). Bars show mean ± SEM, dots represent individual mice, n = 4/group. Asterisks indicate statistical significance compared to the saline group, where ****P < 0.0001 using 2-way ANOVA with Tukey’s multiple comparisons test. Splenocytes (4 wk after the third immunization) and cells from lung homogenates (4 wk after infection with M.tb HN878) were cultured and stimulated with ID93 *ex vivo* and evaluated for CD4^+^ T cell responses by intracellular cytokine staining flow cytometry. ID93-stimulated polyfunctional (expressing IFN-γ, IL-2, TNF, or a combination of these cytokines) CD4^+^ T_H_1 T cells (D) in the spleen post-immunization, and (E) in the lung post M.tb HN878 challenge. Bars show mean ± SEM, n = 4/group. Asterisks indicate statistical significance compared to the saline group, where **P < 0.01 and ****P < 0.0001 using 1-way ANOVA with Tukey's multiple comparisons test.

As antibody-mediated responses could play a role in protection against M.tb infection, we next evaluated humoral vaccine immunogenicity in mice that were immunized with BCG, EmT4™ only, and ID93+EmT4™ and then challenged with M.tb HN878 (Fig. 7A–C) and M.tb H37Rv (Fig. S5A–C). Serum samples were collected 4 weeks after the third immunization and 4 wk post-M.tb HN878 or M.tb H37Rv infection and assessed for an ID93-specific humoral immune response. At both timepoints, mice immunized with ID93+EmT4™ demonstrated the most robust ID93-specific total IgG, IgG1, and IgG2c antibody response. Interestingly, BCG immunization also induced a strong ID93-specific total IgG, IgG1, and IgG2c antibody response post M.tb H37Rv infection compared to the saline group (Fig. S5A–C). These data indicate that immunization with ID93+EmT4™ induces a high magnitude of ID93-specific humoral response both post-immunization and post-M.tb HN878 and M.tb H37Rv infection.

Next, to determine vaccine mediated ID93-stimulated CD4^+^ T_H_1 immune responses, splenocytes were isolated 4 wk post-third immunization and lung cells were isolated four weeks post-M.tb HN878 and M.tb H37Rv infection, and restimulated *ex vivo* with the ID93 fusion antigen. These cells were then stained and evaluated by flow cytometry for CD4^+^ T_H_1 responses (Fig. 7D, E and Fig. S5D, E). Post-immunization, the magnitude of polyfunctional ID93-stimulated CD4^+^ T cells expressing 2 or more cytokines (IFN-γ, IL-2, and TNF-α, or a combination of these cytokines) was significantly increased in the lymphocytes isolated from splenocytes of mice immunized with ID93+EmT4™ compared to mice immunized with BCG or EmT4™ only (Fig. 7D and Fig. S5D). Post-M.tb HN878 infection, the magnitude of ID93-stimulated *ex vivo* polyfunctional CD4^+^ T_H_1 T cells was significantly higher in the lymphocytes isolated from lung homogenates of mice immunized with ID93+EmT4™ compared to mice immunized with BCG or EmT4™ only (Fig. 7E). However, post-M.tb H37Rv infection, there is only a trending increase in the magnitude of ID93-stimulated polyfunctional CD4^+^ T_H_1 T cells in the lymphocytes isolated from lung homogenates of mice immunized with ID93+EmT4™ (Fig. S5E). These data indicate that immunization with ID93+EmT4™ is effective in driving robust ID93-stimulated cellular adaptive immune responses both post-immunization and post-M.tb HN878 infection.

### Prophylactic pulmonary protection

**Figure 8.**
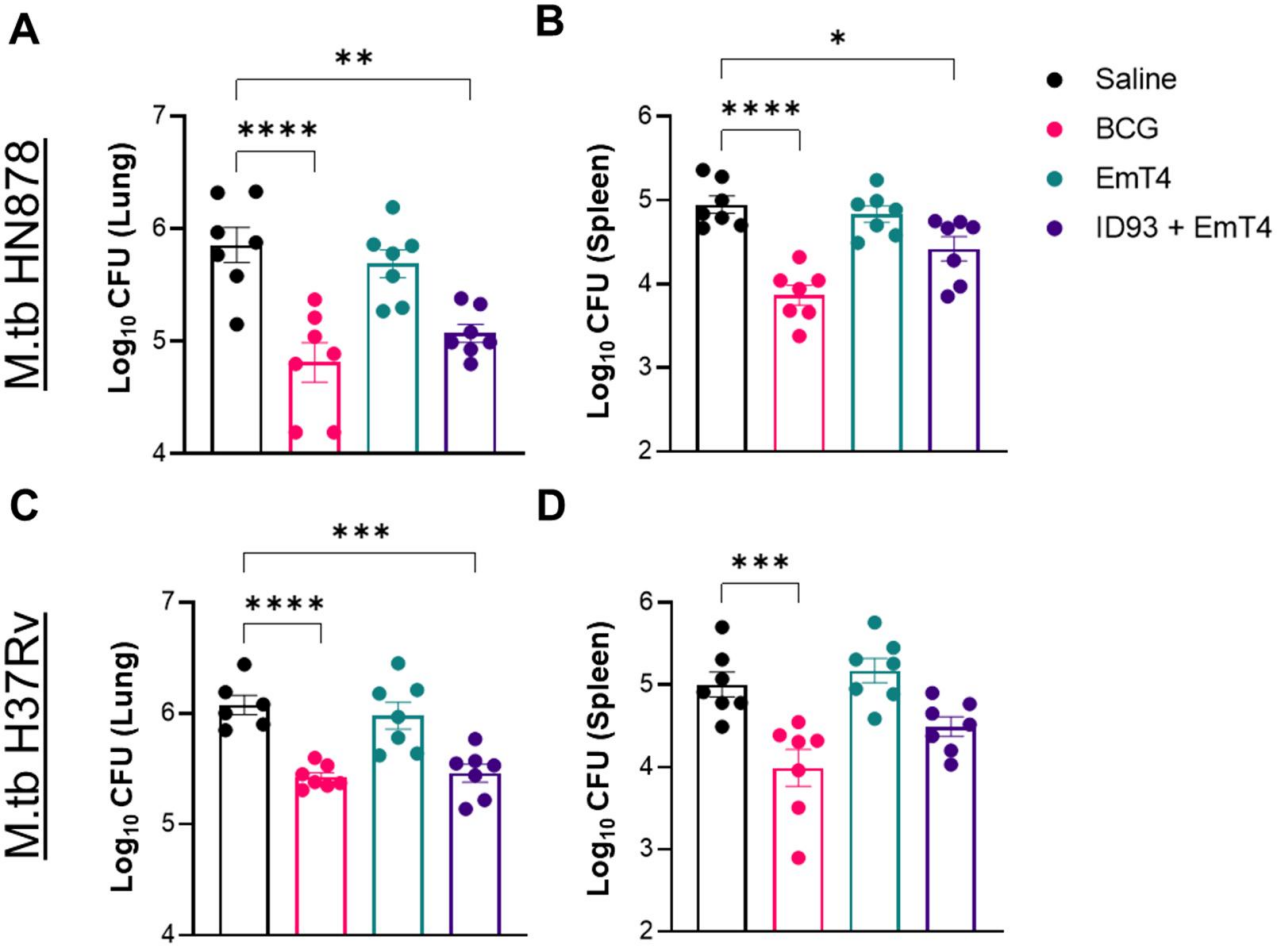
ID93+EmT4™ provides prophylactic pulmonary protection against 2 M.tb isolates at 4 wk post-challenge. Cohorts of mice receiving saline, BCG, EmT4™ or ID93+EmT4™ vaccination regimens were then challenged with a LDA of either M.tb HN878 (A-B) or M.tb H37Rv (C, D) 4 wk after the third immunization. Bacterial burden was assessed by colony forming units (CFU) in lung (A, C) and spleen (B, D) organ homogenates 4 wk after challenge. Bars show the mean ± SEM and dots represent individual mice (n = 5-7/group). Asterisks indicate statistical significance compared to the saline group, where *P < 0.05, **P < 0.01, ***P < 0.001, and ****P < 0.0001 using 1-way ANOVA with Dunnett’s multiple comparisons test.

Four weeks following aerosol M.tb HN878 or M.tb H37Rv challenge, a cohort of seven mice per group were assessed for bacterial burden within the lung and spleen as the primary endpoints for protective efficacy. Immunization with ID93+EmT4™ induced significant prophylactic protection against M.tb HN878 as observed by a reduction in bacterial burden within the lung and spleen compared to the saline group (Fig. 8A, B). Significant vaccine induced prophylactic pulmonary protection against M.tb H37Rv was limited to the lung compared to the saline group (Fig. 8C). Although a protective trend against M.tb H37Rv was observed in the spleen of the ID93+EmT4™ immunized mice, it did not reach statistical significance (Fig. 8D). As expected from our positive control, BCG provided pulmonary and dissemination protection against both M.tb HN878 and M.tb H37Rv challenge as observed by a significant decrease in bacterial burden in the lung and spleen, respectively.

### Protection in young and aged immunocompromised Beige mice

**Figure 9.**
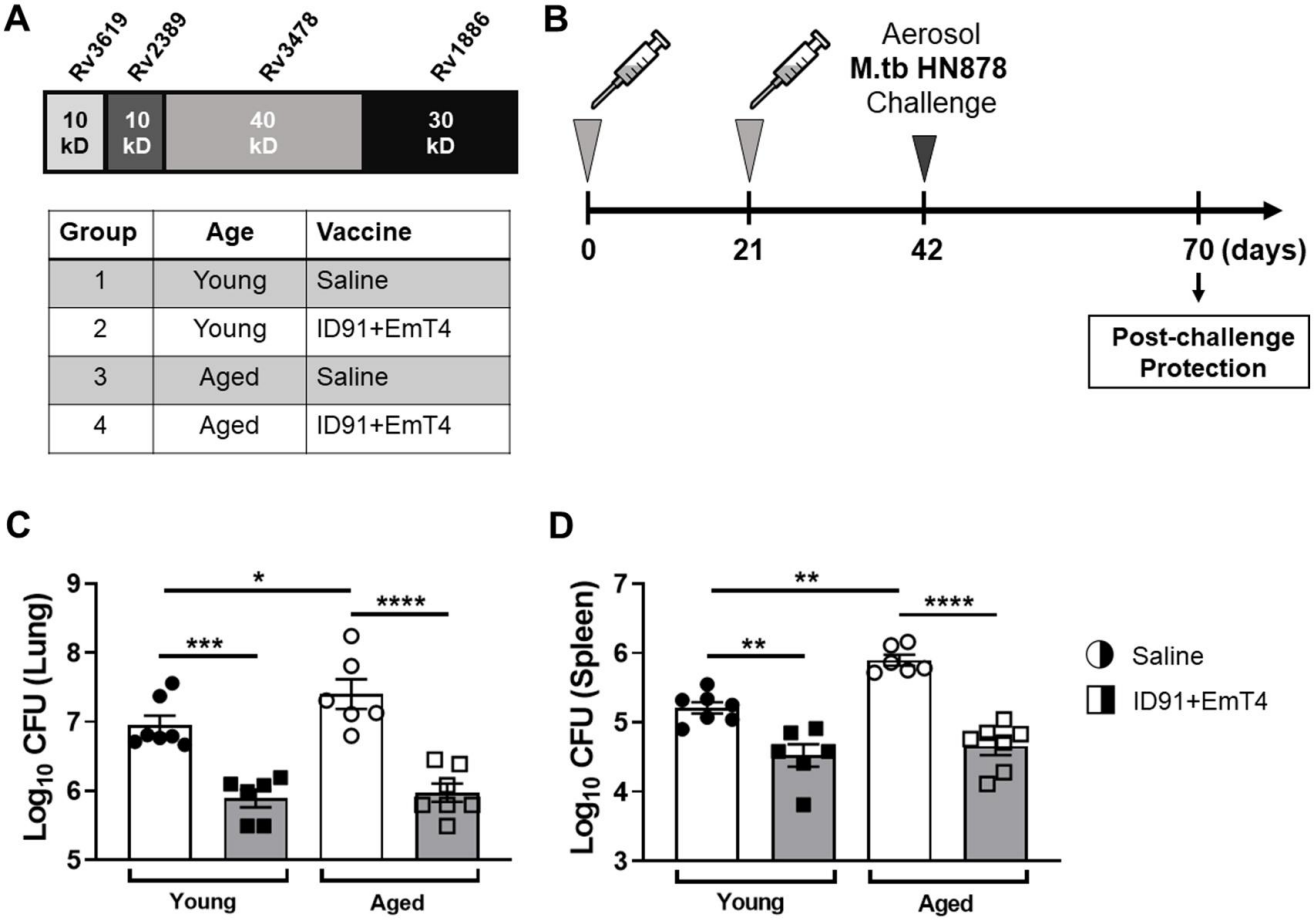
ID91+EmT4™ vaccination provides prophylactic protection against M.tb HN878 in both young and aged Beige mice. (A) The 4 M.tb proteins comprising the ID91 antigen, and immunizations received by each group. (B) Timeline of immunizations and aerosol M.tb HN878 challenge. Experimental samples were collected at D70 (4 wk post M.tb HN878 challenge). Bacterial burden in (C) lung and (D) spleen organ homogenates of young (6 to 8 wk) and aged (20 mo) Beige mice immunized with either saline (circles) or ID91+EmT4 (squares and grey bar) post M.tb HN878 challenge. Bars show mean ± SEM, dots represent individual mice, n = 6-7/group. Asterisks indicate statistical significance compared to the saline group, where *P < 0.05, **P < 0.01, ***P < 0.001, and ****P < 0.0001 using 1-way ANOVA with Dunnett’s multiple comparisons test. Data representative of 1 experiment.

To demonstrate the broad applicability of the EmT4™ adjuvant we tested it in 2 separate escalating contexts: (1) partnered with a different antigen (ID91) and (2) in the condition of immunocompromised Beige (decreased cytotoxic activity) aged mice. Furthermore, it is well documented that male mice are less well protected with vaccination than similar female cohorts.^60–62^ These 3 escalating factors were used in an evaluation of EMT4’s ability to elicit protective immune responses for the most vulnerable populations needing vaccines globally. In this study EmT4™ was formulated with next-generation preclinical TB vaccine candidate ID91, which is composed of M.tb proteins Rv3619 (esxV; ESAT6-like protein), Rv2389 (RpfD), Rv3478 (PPE60), and Rv1886 (Ag85B)^38^^,^^49^ (Fig. 9A). We hypothesized that T_H_1-skewed vaccine-induced immunity could be shown for EmT4™ partnered with different antigens. In addition, we wanted to address the population dynamics of mycobacterial infections disproportionately affecting immunocompromised individuals (genetic or acquired), including aged populations undergoing immunosenescence. In order to do this, we leveraged the Beige mouse as a representative model susceptible to mycobacterial infections as previously described by our group.^46^ Our previous work has demonstrated that ID91 administered only twice demonstrated efficacy, compared to the 3 dose regimen required for ID93 with other commercial TLR4 agonists.^38^ This same rigor was tested here for EmT4™ delivered twice with ID91.

To evaluate the performance of ID91+EmT4™ in Beige mice, both young (6 to 8 wk) and aged (20 mo) animals were immunized with either saline or ID91+EmT4™ 2 times, 3 wk apart. Three weeks post-second immunization, mice were aerosol challenged with M.tb HN878 and vaccine induced protection was assessed 4 wk post-infection as shown in the experimental timeline (Fig. 9B). Both young and aged Beige mice immunized with ID91+EmT4™ demonstrated robust ID93-specific total IgG, IgG1, and IgG2c antibody responses 4 wk post M.tb HN878 infection (Fig. S6A–C). Furthermore, immunization with ID91+EmT4™ induced significant prophylactic protection against M.tb HN878 in both young and aged Beige mice as observed by a reduction of bacterial burden in the lung (Fig. 9C) and spleen (Fig. 9D) compared to the saline control group. As expected, we observed a significant increase in bacterial burden in both the lung and spleen of aged Beige mice mock-immunized with saline, compared to young Beige mice (Fig. 9C, D), indicating an increased susceptibility with age. These data indicate that immunization with ID91+EmT4™ provides prophylactic protection against M.tb HN878 in immunocompromised Beige mice and can help overcome age-related immunosenescence.

## Discussion

The current TB vaccine pipeline is highly diverse, and includes live attenuated, whole cell inactivated, viral vector, and subunit vaccine candidates.^15^^,^^63^^,^^64^ Subunit TB vaccines are strong contenders as they are designed using specific antigens that can target all stages of M.tb infection, while also being safe for use in immunocompromised populations.^65^^,^^66^ However, the limitation for subunit vaccines in general is the low immunogenicity of most proteins by themselves. Therefore, subunit vaccines rely on adjuvants to adequately stimulate and activate the immune response to the protein antigens, enhance epitope coverage, increase immune breadth, and drive durable memory.^67–70^ Development and characterization of new adjuvants for vaccines against M.tb has several potential advantages including: (1) improved vaccine efficacy- adjuvants can amplify the immune response to vaccine antigens, which can result in improved protection against disease; (2) increased vaccine coverage- adjuvants can allow for vaccine dose sparing, which can make vaccines more affordable and accessible; (3) enhanced safety- next generation adjuvants can allow for the use of safer, more purified vaccine antigens, reducing the risk of adverse reactions; and (4) broader immunity- adjuvants may enhance the breadth of immunity which can be important for diseases like TB.

There are several candidates in the clinical TB pipeline formatted as subunit protein-adjuvants including: (i) M72 fusion protein + AS01, a liposomal formulation of MPL (TLR4 agonist) and the saponin QS-21,^71–74^ (ii) ID93 fusion protein + GLA-SE (TLR4 agonist),^36^^,^^75^^,^^76^ (iii) H56 antigen + antibacterial peptide (KLKL(5)KLK) with synthetic oligodeoxynucleotide (IC31, TLR9 agonist^77–79^) (iv) Ag85A-ESAT6-CFP10 fusion antigen + DEAE-dextran core formulated with cytosine–phosphate–guanine (CpG) oligodeoxynucleotides (GamTBVac; TLR9 agonists^80^^,^^81^) and (v) Ag85b-ESAT6-CFP10 fusion antigen (AEC) + BCG-derived unmethylated CpG and aluminum salt (BC02; TLR9 agonist^82^^,^^83^) according to the Tuberculosis Vaccine Initiative Pipeline. Excitingly, recent Phase IIB trial results of the M72 + AS01 vaccine demonstrated 49.7% protection against M.tb after 3 yr of follow-up,^74^ suggesting subunit + adjuvant strategies are a viable approach for POD indications. Adjuvant use can also span many discrete pathogens, for example, the AS01 adjuvant system has been extended for use in the FDA approved shingles vaccine “Shingrix™” and is approved for use in malaria as the Mosquirix™ vaccine.^72^

Here we evaluated the ability of a novel, synthetic TLR4 agonist vaccine adjuvant, EmT4™, to induce T_H_1-biased proinflammatory immunity and prophylactic protection against aerosol challenge with M.tb HN878. We observed EmT4™ induce a dose-dependent activation of BMDM and BMDCs, innate hallmarks for the initiation of a proinflammatory immune response. Clinical TB vaccine candidate antigen ID93 paired with EmT4™ induced robust proinflammatory cytokines and chemokines that were detectable in the sera of immunized mice post-boost and remained preferentially higher than control cohorts post-aerosol challenge with 2 different M.tb strains. Vaccination with ID93+ EmT4™ was prophylactically protective when measured by bacterial burden against both M.tb HN878 and M.tb H37Rv challenge, demonstrating the cross lineage coverage. This protection is likely in part due to the robust antigen-specific humoral (IgG) and cellular (polyfunctional T_H_1 CD4^+^ T cells) immunity induced post vaccination. Importantly, we observed that this vaccine-induced protection extended across use with separate antigens, ID93 and ID91, and in the context of both immune competent C57BL/6 mice as well as immunocompromised young and aged Beige mice. Together these data indicate that immunization with EmT4™ augments prophylactic immunogenicity and resulting antigen-specific immunity confers pulmonary protection against M.tb. EmT4™ represents a novel TLR4 agonist-based adjuvant that enhances the proinflammatory immune response to co-formulated antigen presumably by downstream signaling through MyD88 and TRIF/TRAF pathways similar to other synthetic TLR4 agonists. This synthetic formulation of MPL provides a source of stable and uniform adjuvant product with diverse applications. EmT4™ joins other TLR4 adjuvants such as AS01 (GSK plc, Brentford, United Kingdom), and GLA-SE (AAHI, Seattle Washington, USA), as effective adjuvants for use with M.tb subunit vaccines, complementing the suite of similarly proinflammatory adjuvants including other TLR agonists such as the TLR9 agonists IC31 (Valneva Austria GmbH, Vienna, Austria), CpG (Dynavax Technologies, Emeryville, California, USA) and BC02 (BCG CpG DNA compound adjuvant system 02 plus Al(OH)_3_).^84^ Therefore, we recommend EmT4™ as a low-cost candidate for inducing T_H_1-driven immunity against M.tb and suggest that it should be leveraged to help diversify and provide depth to the TB vaccine pipeline.

## Supplementary Material

vlaf014_Supplementary_Data

## Data Availability

The data underlying this article are available in the article and in its online supplementary material, or are available on reasonable request to the corresponding author.
