# Supplementary material for: Characterizing TLR4 agonist EmT4™ as an anti-Mycobacterium tuberculosis vaccine adjuvant
Source: Immunohorizons. 2025 Apr 24;9(6):vlaf014. doi: 10.1093/immhor/vlaf014 (PMC12032397; doi:10.1093/immhor/vlaf014)
Supplement: vlaf014_Supplementary_Data [file vlaf014_supplementary_data.zip › IMMHOR-23-00037-s02.pdf]

Characterizing TLR4 agonist EmT4™ as an anti-*Mycobacterium tuberculosis* vaccine adjuvant

Sasha E. Larsen[*#], Maham Rais[*#], Valerie A. Reese[*], Debora Ferede[*], Tiffany Pecor[*], Suhavi Kaur[*], Deepika Nag[*], Thomas Smytheman[*], Sean A. Gray[†], Darrick Carter[†], Susan L. Baldwin[*##], Rhea N. Coler[*,‡,§,##]

Supplemental Figures

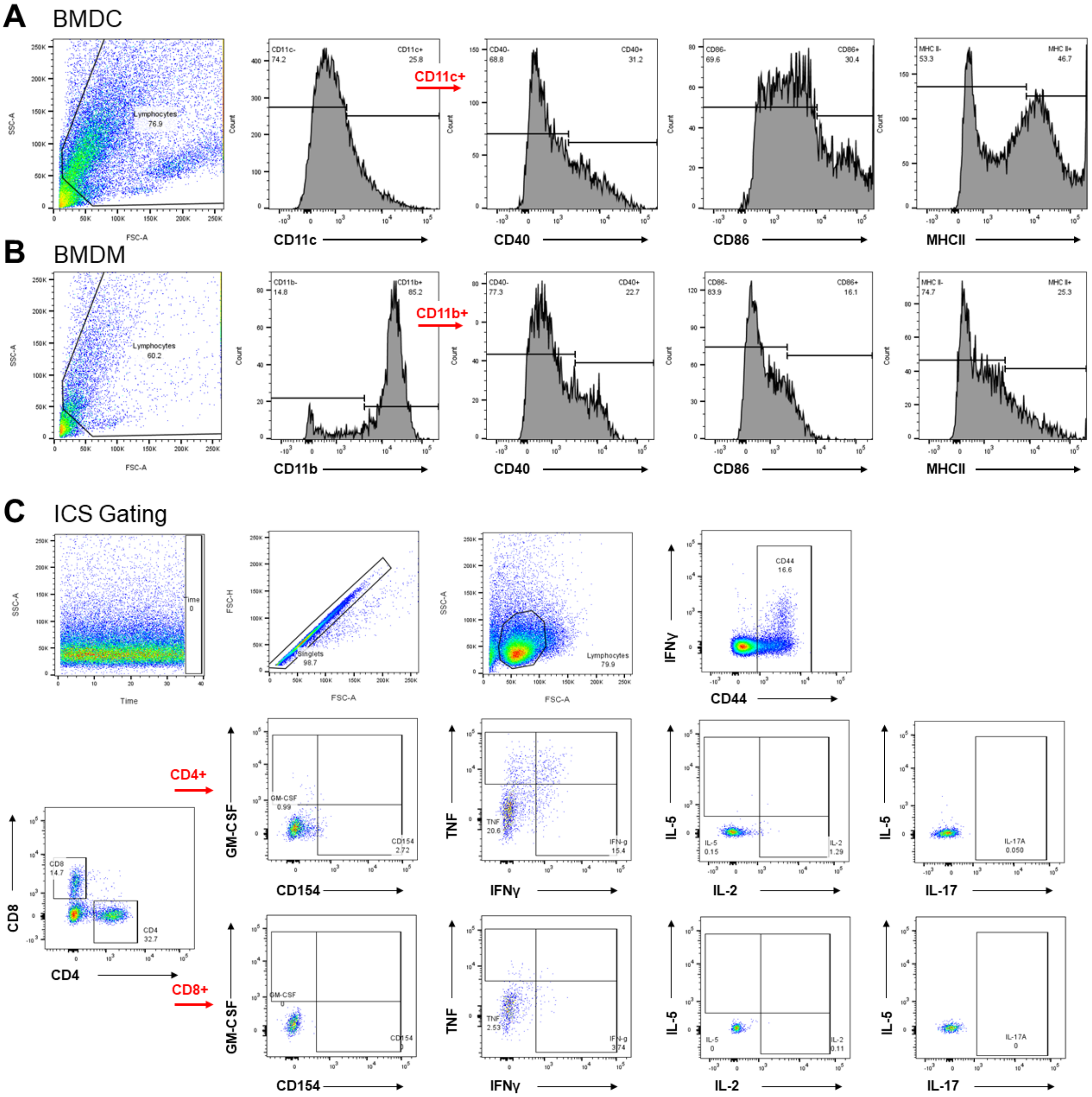

Supplementary Figure 1. Activation of BMDC and BMDM and Intracellular cytokine staining (ICS) flow cytometry gating scheme. Example of gating scheme for (**A**) CD11c+ BMDC expressing CD40, CD86, and MHCII cell surface markers, and (**B**) CD11b+ BMDM expressing CD40, CD86, and MHCII cell surface markers (Paired with Figure 2). (**C**) Example of ICS gating scheme for CD44+, CD4+, and CD8+ T cells expressing GM-CSF, CD154, T_H1 (IFN-γ, TNF-α, IL-2), T_H2 (IL-5), and T_H17 cytokines (IL-17A) in splenocytes that were restimulated *ex vivo* with ID93 four weeks post third immunization (Paired with Figure 6).

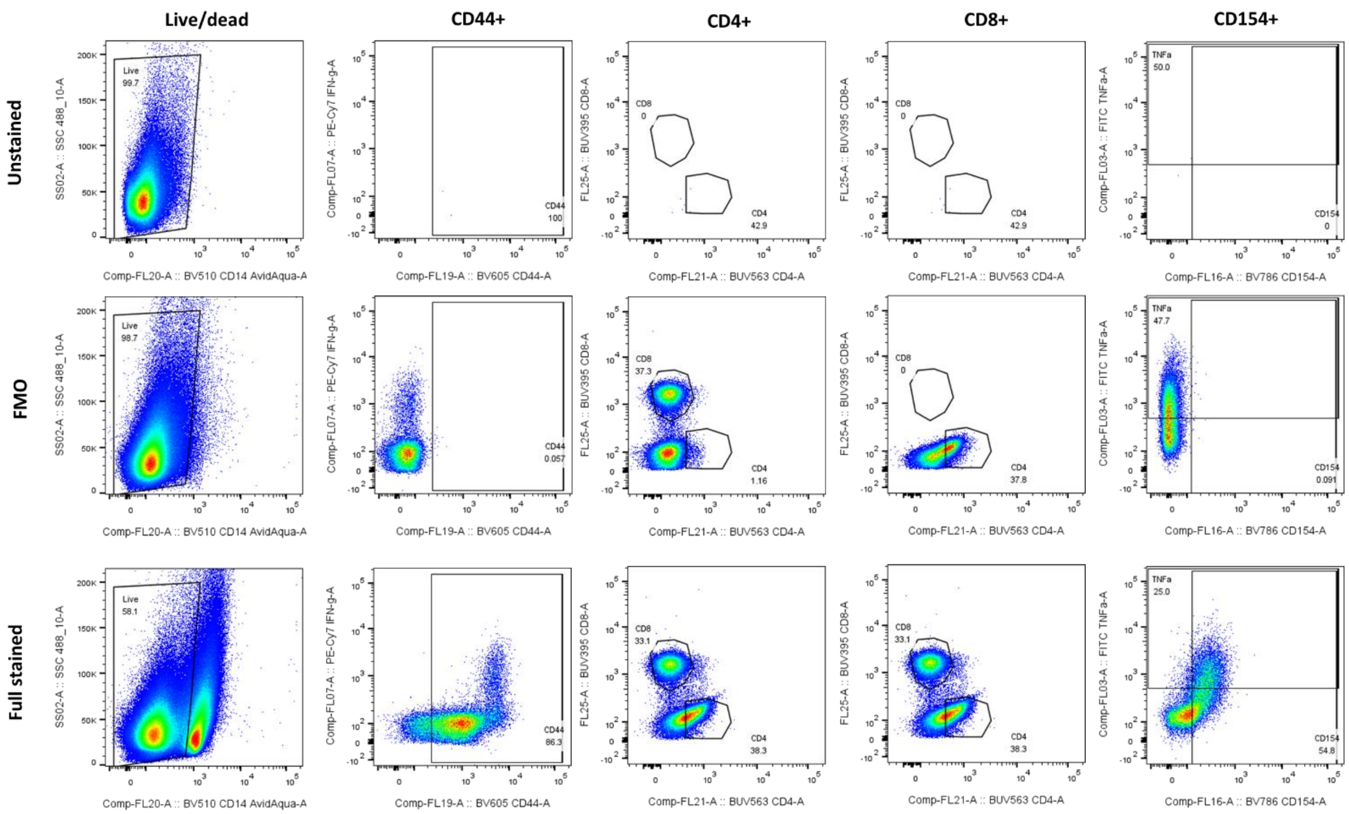
Live/dead
CD44+
CD4+
CD8+
CD154+
Unstained
FMO
Full stained
Comp-FL20-A :: BV510 CD14 AvidAqua-A
SS02-A :: SSC 488_10-A
Comp-FL19-A :: BV605 CD44-A
Comp-FL07-A :: PE-Cy7 IFN-g-A
Comp-FL21-A :: BUV563 CD4-A
FL25-A :: BUV395 CD8-A
Comp-FL16-A :: BV786 CD154-A
Comp-FL03-A :: FITC TNFa-A

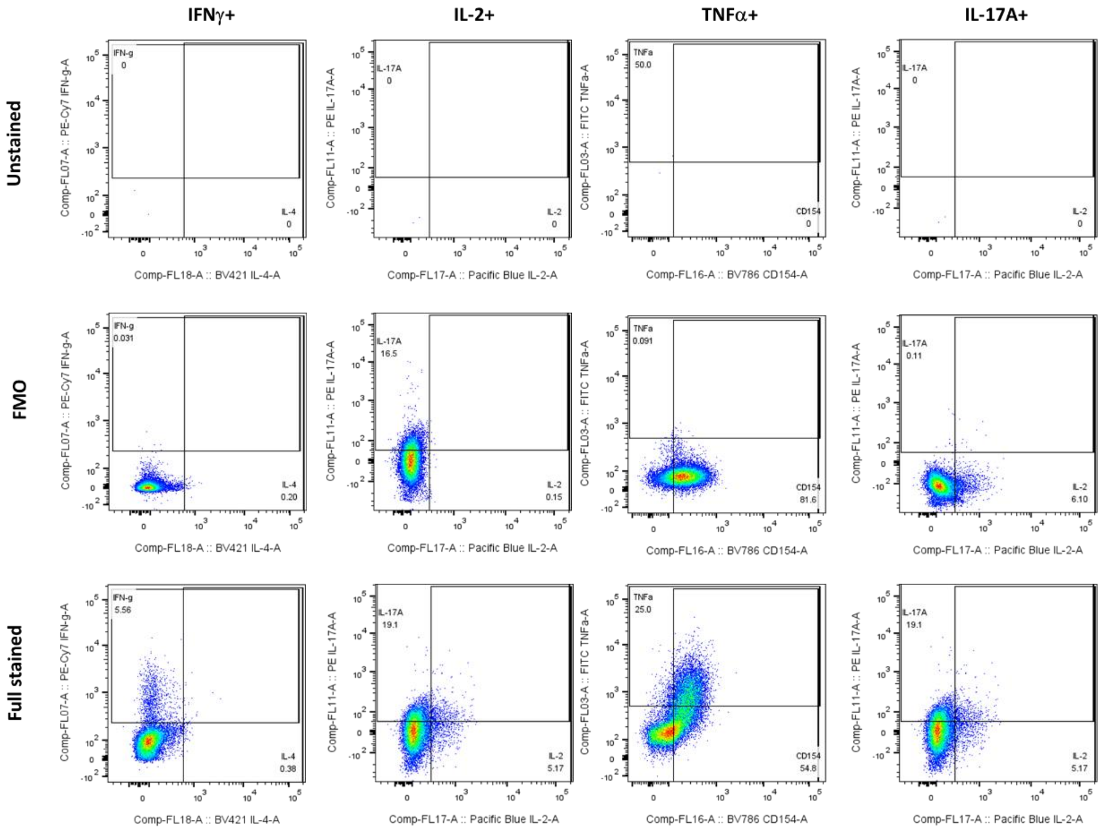
IFNγ+
IL-2+
TNFα+
IL-17A+
Unstained
FMO
Full stained
Comp-FL18-A :: BV421 IL-4-A
Comp-FL07-A :: PE-Cy7 IFN-g-A
Comp-FL17-A :: Pacific Blue IL-2-A
Comp-FL11-A :: PE IL-17A-A
Comp-FL16-A :: BV786 CD154-A
Comp-FL03-A :: FITC TNFa-A

16

Supplementary Figure 2. Non-Stained Cells, Fluorescence Minus One (FMO) Control, and Full Stained Cells for Staining Panel. Representative flow cytometry plots demonstrating the gating strategy for Live/Dead, CD44, CD4, CD8, CD154, IFN-γ, IL-2, TNF-α, and IL-17a staining. Non-stained cells serving as a negative control to establish the baseline fluorescence. FMO control plots showing cells stained with all antibodies except one, to accurately define the background fluorescence and set gating thresholds for each marker. Full stained cells demonstrating the complete staining profile.

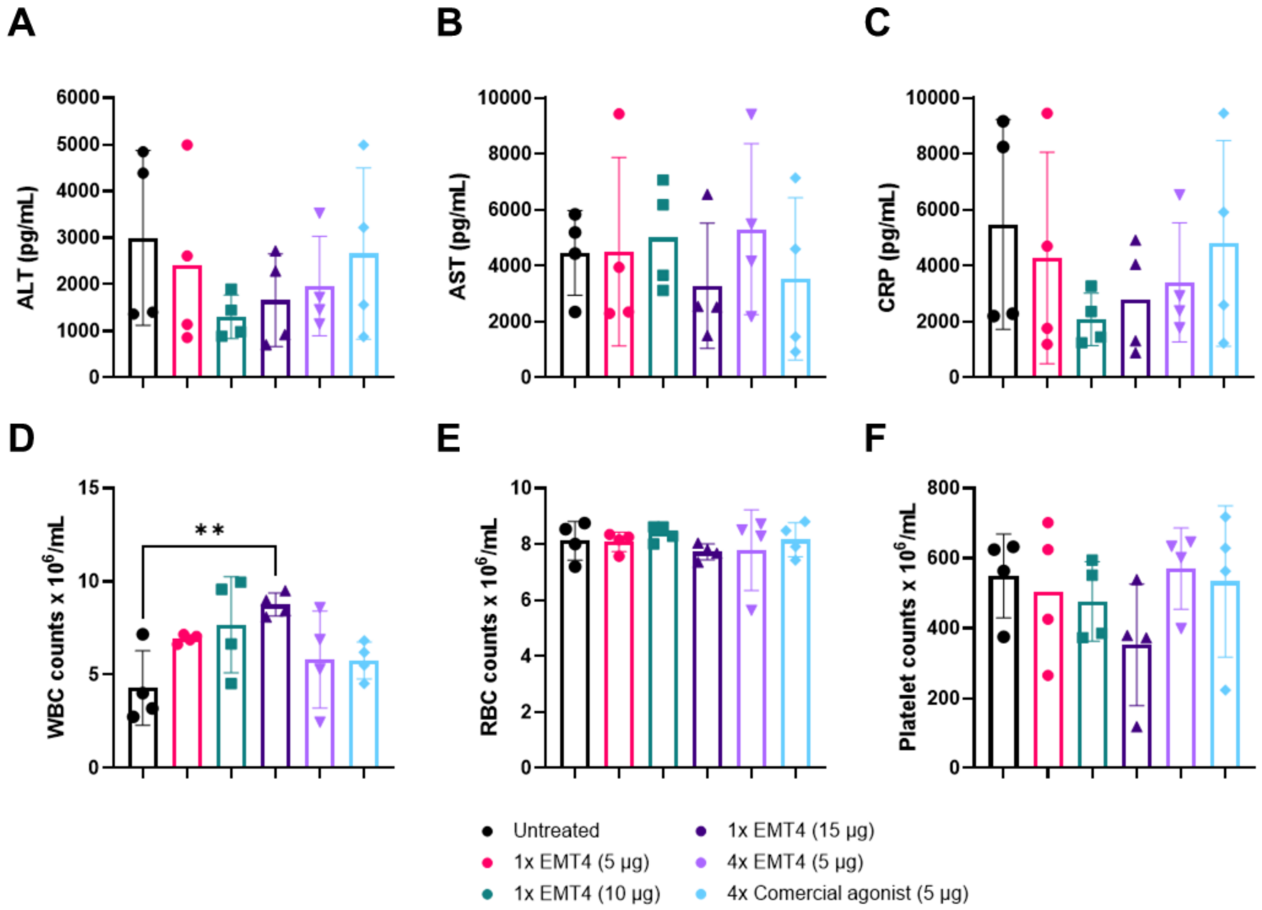

Supplemental Figure 3. Whole blood evaluation of EMT4™ tolerability in the mouse with repeated and escalated dosing. C57BL/6 mice were left untreated (black), given a single immunization with 5 µg (pink), 10 µg (teal), or 15 µg (dark purple) of EMT4™ or in separate

cohorts given 4 consecutive immunizations with 5 µg of EMT4™ (light purple), or 5 µg of a commercial TLR4 agonist (blue). Plasma collected on day 12 post study start was used in commercial ELISAs to enumerate the levels of (**A**) ALT, (**B**) AST and (**C**) CRP from each cohort. Whole blood collected on day 12 post study start was evaluated for (**D**) whole blood cell counts, (**E**) red blood cell counts and (**F**) platelet counts. Treated cohorts were individually compared to untreated using a two-way ANOVA with Dunnetts multiple comparisons, significant differences are denoted where ** $p < 0.01$.

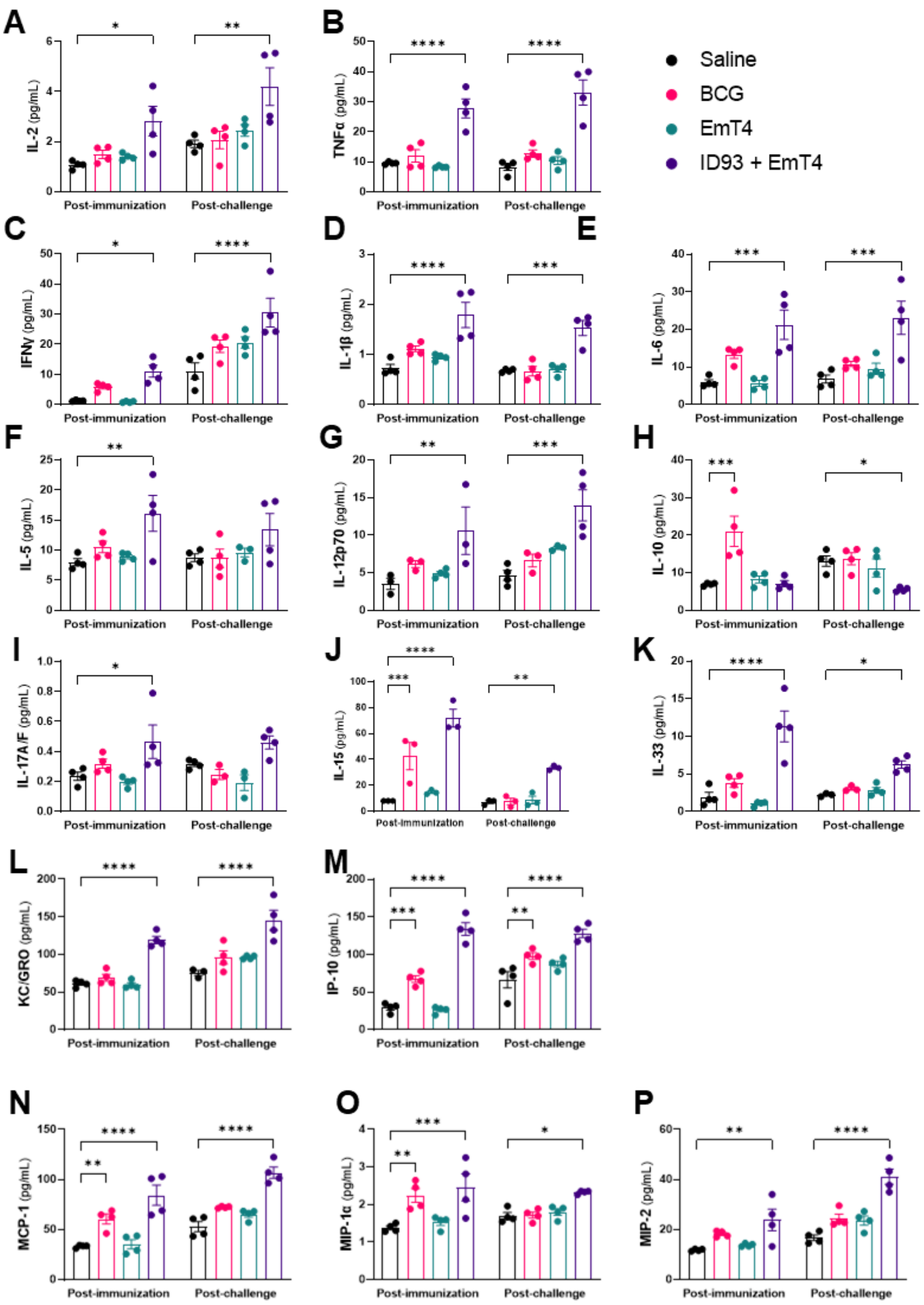

Supplementary Figure 4. Vaccination with ID93+EmT4™ induces robust circulating cytokine and chemokine responses post-immunization and post-M.tb H37Rv challenge. Systemic cytokine and chemokine levels were measured in serum samples collected 4 weeks after

the third immunization and 4 weeks after infection with M.tb H37Rv. Concentration of (**A**) IL-2, (**B**) TNF-α, (**C**) IFN-γ, (**D**) IL-1β, (**E**) IL-6, (**F**) IL-5, (**G**) IL-12p70, (**H**) IL-10, (**I**) IL-17A/F, (**J**) IL-15, and (**K**) IL-33 pg/mL post-immunization (left) and post-challenge (right). Concentration of (**L**) KC/GRO, (**M**) IP-10, (**N**) MCP-1, (**O**) MIP-1α, and (**P**) MIP-2 (measured in pg/mL) post boost immunization (left) and post infection (right). Bars show mean ± SEM, dots represent individual mice, n = 4/group. Asterisks indicate statistical significance compared to the saline group, where $*p < 0.05$, $**p < 0.01$, $***p < 0.001$, and $****p < 0.0001$ using two-way ANOVA with Tukey's multiple comparisons test (Paired with Figures 4 and 5).

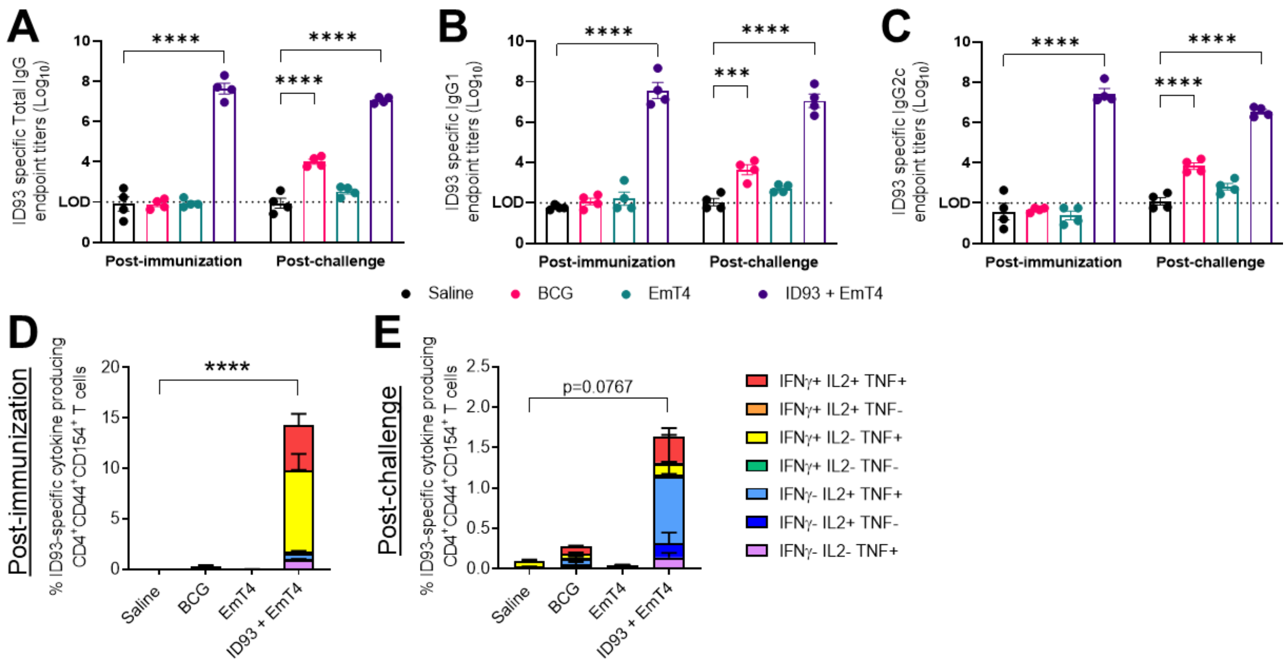

Supplementary Figure 5. Vaccination with ID93+EmT4™ drives ID93 antigen-specific humoral and cellular immune responses post-immunization and post-M.tb H37Rv infection. Serum samples were collected 4 weeks after the third immunization and 4 weeks after infection with M.tb H37Rv and were evaluated for: ID93 antigen-specific (**A**) Total IgG, (**B**) IgG1, and (**C**) IgG2c responses. Log_{10} endpoint titer (EPT) is shown (LOD = limit of detection of the assay). Bars show mean ± SEM, dots represent individual mice, n = 4/group. Asterisks indicate statistical significance compared to the saline group, where $***p < 0.001$ and $****p < 0.0001$ using two-way ANOVA with Tukey's multiple comparisons test. Splenocytes (4 weeks after the third immunization) and cells from lung homogenates (4 weeks after infection with M.tb H37Rv)

were cultured and stimulated with ID93 *ex vivo* and evaluated for CD4+ T cell responses by intracellular cytokine staining flow cytometry. ID93-specific polyfunctional (expressing IFN-γ, IL-2, TNF or a combination of these cytokines) CD4+ T_H1 T cells (**D**) post-immunization and (**E**) post M.tb H37Rv challenge. Bars show mean ± SEM, n = 4/group. Asterisks indicate statistical significance compared to the saline group, where ****$p < 0.0001$ using one-way ANOVA with Tukey's multiple comparisons test (Paired with Figure 6).

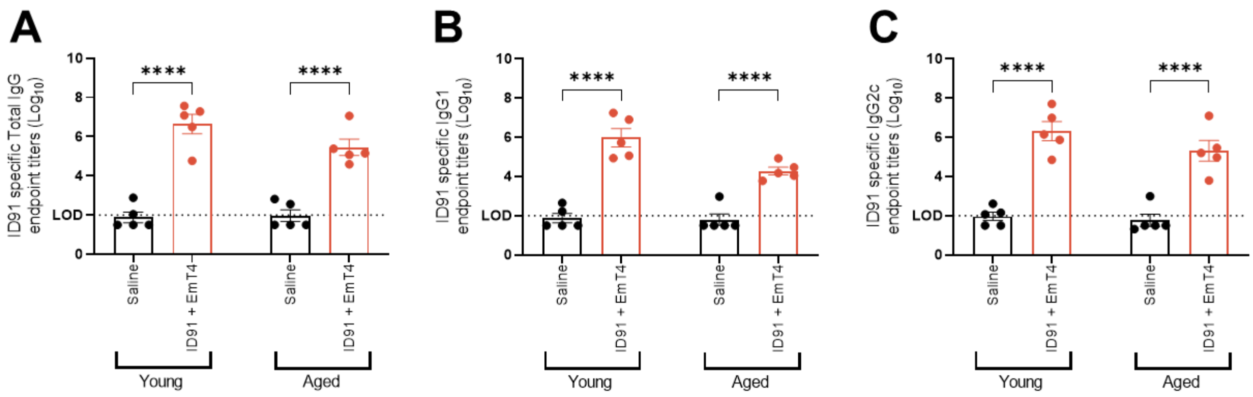

Supplementary Figure 6. Vaccination with ID91+EmT4™ drives ID91 antigen-specific humoral immune responses post M.tb HN878 infection in both young and aged Beige mice. Serum samples were collected from young and aged Beige mice 4 weeks after infection with M.tb HN878 and were evaluated for: ID93 antigen-specific (**A**) Total IgG, (**B**) IgG1 and (**C**) IgG2c responses. Log_{10} endpoint titer (EPT) is shown (LOD = limit of detection). Bars show mean ± SEM, dots represent individual mice, n = 4/group. Asterisks indicate statistical significance compared to the saline group, where ****$p < 0.0001$ using two-way ANOVA with Dunnett's multiple comparisons test (Paired with Figure 8).
