# Supplementary material for: Characterizing TLR4 agonist EmT4™ as an anti-Mycobacterium tuberculosis vaccine adjuvant
Source: Immunohorizons. 2025 Apr 24;9(6):vlaf014. doi: 10.1093/immhor/vlaf014 (PMC12032397; doi:10.1093/immhor/vlaf014)
Supplement: vlaf014_Supplementary_Data [file vlaf014_supplementary_data.zip › IMMHOR-23-00037-s01.docx]

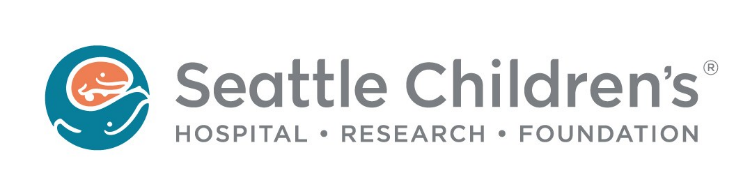


**ImmunoHorizons**

**To: Editorial Staff**

**Re: New Manuscript Submission**

27 November 2024

Dear *ImmunoHorizons* Editorial Staff,

We are pleased to share our manuscript “Characterizing TLR4 agonist EmT4™ as an anti-*Mycobacterium tuberculosis* vaccine adjuvant” for consideration as a primary full-length research article in *ImmunoHorizons.* We believe this work adds valuable information about a novel vaccine adjuvant targeting the TLR-4 pathway. In this work we evaluate the essential readouts of immunogenicity: intracellular cytokine production in response to antigenic stimulation, humoral immunity, cytokine production and protective efficacy in an *in vivo* mouse model of *Mycobacterium tuberculosis*. We’ve even examined the benefits of using this vaccine in the context of aged mice, and demonstrate it alleviates immunosenescence. We have made significant edits in response to prior reviewer comments, including the addition of tolerability data to this manuscript since its first submission to the *Journal of Immunology* in 2023 and we believe this enhances the work considerably. To our knowledge there is no existing work describing this adjuvant, its immunogenic properties or induced protection in the context of a pathogenic model. Although this work was completed in the context of our pathogen of interest, *M. tuberculosis,* the immunogenicity and use of this adjuvant may be widely applicable. Importantly, we feel that the work described in this manuscript will be of interest to the readership of your journal, meeting the scope of “characterization of novel reagents” and could fit into the “Infectious Disease” publication section.

We have identified several potential reviewers for your consideration and have listed their details below. We are respectfully requesting the exclusion of any reviewer who currently works at Access to Advanced Health Institute (AAHI, formerly known as the Infectious Disease Research Institute), of Seattle, WA. We look forward to hearing from you about the appropriateness of our article for the *ImmunoHorizons* and the prospective review process.

Warm Regards,

**Sasha E. Larsen, Ph.D.**

*Co-First Author

Research Scientist IV, Supervisor

Seattle Childrens Research Institute

Center for Global Infectious Disease Research

**Maham Rais, Ph.D.**

*Co-First Author

Postdoctoral Scientists

Seattle Children’s Research Institute

Center for Global Infectious Disease Research

&

**Rhea N. Coler, M.Sc., Ph.D.**

**Corresponding Author

Professor, Department of Pediatrics, University of Washington School of Medicine

Senior Investigator, Center for Global Infectious Disease Research (CGIDR)

Adjunct Professor, Department of Global Health, University of Washington

Member, Brotman Baty Institute

206-884-3336 OFFICE

Rhea.Coler@seattlechildrens.org

We respectfully present potential reviewers for your consideration including their contact information and relevant recent publication related to our areas of expertise in this manuscript submission. We have not published nor shared funding with any of these individuals.

| **Jose Perez-Casal**  Vaccine and Infectious Disease Organization, Saskatoon, Canada  [jose.perez-casal@usask.ca](mailto:jose.perez-casal@usask.ca)  PMID: 36774333 | **Xiang Luo**  Joint Laboratory for Translational Cancer Research of Chinese Medicine of the Ministry of Education of the People's Republic of China  [liao@gzucm.edu.cn](mailto:liao@gzucm.edu.cn) [luoxiang@gzucm.edu.cn](mailto:luoxiang@gzucm.edu.cn)  PMID: 36760743 | **Mangala Rao**  Laboratory of Adjuvant & Antigen Research, U.S. Military HIV Research Program, WRAIR  [mrao@hivresearch.org](mailto:mrao@hivresearch.org)  PMID: 33165871 |
| --- | --- | --- |
| **Francesco Peri**  Department of Biotechnology and Biosciences, University of Milano-Bicocca, Italy  [francesco.peri@unimib.it](mailto:francesco.peri@unimib.it)  PMID: 36728697 | **Robert K Ernst**  Department of Microbial Pathogenesis, University of Maryland School of Dentistry, Baltimore, Maryland, USA  [rkernst@umaryland.edu](mailto:rkernst@umaryland.edu)  PMID: 28487429 | **Alla Zamyatina**  Department of Chemistry, University of Natural Resources and Life Sciences, Austria  [Alla.zamyatina@boku.ac.at](mailto:Alla.zamyatina@boku.ac.at)  PMID: 35439332 |
